# Clinical Trials with Herbal Products for the Prevention of Dental Caries and Their Quality: A Scoping Study

**DOI:** 10.3390/biom9120884

**Published:** 2019-12-17

**Authors:** Robert Ancuceanu, Adriana Iuliana Anghel, Camelia Ionescu, Marilena Viorica Hovaneț, Maria Cojocaru-Toma, Mihaela Dinu

**Affiliations:** 1 Faculty of Pharmacy, Carol Davila University of Medicine and Pharmacy, 020956 Bucharest, Romaniamarilenaviorica@yahoo.com (M.V.H.); mihaela.dinu@umfcd.ro (M.D.); 2 Faculty of Dental Medicine, Carol Davila University of Medicine and Pharmacy, 010221 Bucharest, Romania; camidentro@yahoo.com; 3 Faculty of Pharmacy, Nicolae Testemițanu University of Medicine and Pharmacy, 2025 Chisinau, Moldavia; maria.cojocaru@usmf.md

**Keywords:** dental caries, prevention, clinical trials, herbal, scoping review

## Abstract

It is currently recognized that an injudicious strategy about caries in the last decades has been not only focusing of research mostly in children, but also the narrow focusing on fluoride, because despite sufficient availability of fluoride in water and oral healthcare products, caries levels escalate steadily as people get older and caries remain a main public health issue to be settled. In the last two decades the scientific community intensified efforts of exploring other products for caries prevention, herbal products being one of these approaches. Preliminary evidence indicated that clinical trials for caries prevention with herbal products are heterogeneous in design, quality and products evaluated, we therefore performed a scoping review intended to explore the main characteristics of such clinical trials. From an initial collection of 1986 unique papers from different literature databases, 56 articles satisfied the inclusion and exclusion criteria. The species investigated, dosage forms, study designs, duration of intervention, controls, endpoints, quality of reporting, and risk of bias are discussed. Of the trials reviewed here, 85.71% reported positive results but given the methodological flaws and biases affecting them, it is difficult to conclude on the efficacy of those products based on the studies published thus far.

## 1. Introduction

Dental caries (also known as tooth decay) is regarded as the lifestyle dependent human disease with the highest prevalence in the world [1]. It is not simply “holes in the teeth”, but rather the result of a complex, multifactorial, and dynamic process involving bacteria that produce acids by food fermentation, the acids eroding and dissolving the tooth minerals and hard tissues [2,3]. In the case of children, if not prevented or treated in time, it may have broad negative consequences, affecting the masticatory function, speech, and smile, as well as the quality of life of the little patient [4]. There is accumulating evidence that indicates a potential relationship between the changes in the immune system manifesting slowly in the elderly and the risk of caries development and complications, although this relationship is complex and our knowledge of it is currently very limited and fragmentary [5].

Although the leading model on caries formation has shifted from an emphasis on particular microbial species (“the specific plaque hypothesis”) to a broader involvement of bacterial species and strains (“the ecological plaque hypotheses”), it is generally still accepted that the main causal agents among the mouth bacteria involved in the initiation of the cariogenic process are represented by the mutans streptococci: *Streptococcus mutans* and *Streptococcus sobrinus* [6,7]. In a recent study comparing children with and without caries from Greece (age varying between 3 and 13 years old), *S. mutans* were detected in 66% of the cases, whereas *S. sobrinus* had a frequency of only 11%; the microbes were detected more often and in higher numbers in the children with active caries [8]. In other studies, though, the frequencies of *S. mutans* and *S. sobrinus* in children with caries have been similar [9] or higher for *S. sobrinus* [10], and often both germs have been detected. Preschool [9] and school children [10], as well as children with intellectual disabilities [11] harboring both *Streptococcus species* (*S. mutans* and *S. sobrinus*) tend to have an increased incidence of dental caries than their counterparts harboring *S. mutans* only.

*S. mutans* belongs to a group of bacteria whose pathogenicity is closely related to their capacity of creating biofilms on solid surfaces (such as teeth), developing 3D structures that protect them against antibiotics and other potential aggressors through the interbacterial interactions and an exopolysaccharide-rich matrix [12,13]. This species synthesizes several adhesins with high affinity and specificity for a diversity of constituents of the extracellular matrix (ECM) and other biochemical compounds from the human body or from different species of bacteria [14]. An adhesin protein, located on the cell surface of *S. mutans* and known as spaP, AgI/II, PAc, P1, B, and MSL-1, interacts with a human receptor glycoprotein involved in innate immunity, and when this receptor protein becomes adsorbed upon the surface of the teeth, it will also function as a receptor for the adherence of streptococci such as *S. mutans* [15]. The proteins of the Ag I/II family are involved in the so-called sucrose-independent mechanism of virulence, which is additional to a sucrose-dependent mechanism, involving a series of glucosyltransferases and glucan-binding proteins synthesized by the pathogen [12]. The analysis of mutans streptococci in saliva has been proposed as a tool in assessing the risk of developing caries in individual patients, because a relatively high correlation between bacterial counts in saliva and dental plaque has been shown [16].

Although the mutans streptococci are most widely known for their causative contribution to developing dental caries, a series of data have shown that other microbial species, such as anaerobic *Scardovia wiggsiae*, *Veillonella parvula*, *Streptococcus cristatus*, and *Actinomyces gerensceriae* may also be associated with severe early childhood caries, and the presence of *S. wiggsiae* in particular has been confirmed in cases where *S. mutans* bacteria were not detected [17].

Although acids produced through fermentation of sugars by acidogenic and aciduric bacteria are able to provoke demineralisation of the dental exterior, it has been shown that they are not cariogenic per se, the process of caries development requiring the involvement of proteolytic enzymes active in a low pH environment [18]. Matrix metalloproteinases (MMPs) of human cell origin have been more recently proposed as causally contributing to this process, besides collagenases of bacterial origin [18]. However, although the relevance of these other factors is recognized, it has been argued that in the absence of sugars “the chain of causation is broken” and all other factors are thus mere variables that alter/modify the cariogenic process, but they are not alternative contributors to the effect and thus, it is claimed, is rather misguided to speak about the cariogenic process as a “multifactorial” one [19]. Sucrose is the most cariogenic, but glucose, fructose, and other mono- and disaccharides are also incriminated for their key role in dental caries development (whereas processed food starches have a considerably lower cariogenic potential) [19].

In the last three or four decades the frequency of caries lesions and their severity have decreased in young children, teenagers and adults from a growing number of countries. The factors contributing to this are represented by improved lifestyle habits (increased use of fluoride, especially as toothpaste, some reduced sugar intake has also been claimed) and expanding the frequency of medical check-ups [20]. Despite such improvements, it has been recently stated that irrespective of age, “caries and periodontal diseases are among the most prevalent diseases in mankind” [21], 60–90% of the children and 92–93% of adults from the age of 20 to ages older than 65 years old have dental caries (treated or untreated) [5,22]. In this context improving the prevention and treatment of caries should remain a priority of the health professions.

A slow shift is currently taking place in the dental profession, from a cyclic and repeated restorative approach based on filings to a population and individual prevention approach, restoration being seen rather as a last resort [23]. Nevertheless, it has been acknowledged that the interventions currently in use for the prevention of dental caries are not robustly rooted in scientific evidence, the majority of studies being derived from children and young patients, conducted without considering the caries risk of the subjects, and “there is a lack of evidence for caries preventive methods in adults with increased caries risk” [21,24]. Under the heading “Caries prophylactic agents”, the ATC code (A01AA) developed by the World Health Organization recognizes only a number of four fluoride derivatives: sodium fluoride, sodium monofluorophosphate, olaflur, and stannous fluoride, as well as a number of fluoride combinations. “Antiinfectives and antiseptics for local oral treatment” are grouped under a different subheading of stomatological preparations, because they have a broader range of indications, such as gingivitis, stomatitis, etc. [25], but such products are claimed to be useful inter alia to prevent dental caries [26].

It is nowadays also acknowledged that a misguided strategy in the last decades has been not only the concentration of research efforts mostly on caries in children, but also on the use of fluoride; currently it is uncontested that despite ample availability of fluoride in water and oral healthcare products (toothpaste, oral solutions, etc.), caries levels rise unwaveringly as people get older and the caries remains a key public health issue to be solved [19]. In this context, a certain interest has been manifested (particularly in the last two decades) in the scientific community for exploring other products than fluoride derivatives for caries prevention.

A variety of antibacterial quaternary ammonium compounds or dental materials based on such compounds have been explored [27,28,29,30], as well as polymeric or inorganic nanoscale agents [31]. As for other therapeutic areas [32,33,34,35], mining natural sources such as bee products or medicinal plants and ethnopharmacology data have also been looked upon as a potential way forward. A preliminary search of the published clinical trials carried out for caries prevention with herbal products suggested that there is relatively broad heterogeneity in design, quality, and herbal products evaluated clinically for this purpose. Therefore, a systematic review was considered rather inappropriate; instead, we performed a scoping review whose purpose is generally “to map the body of literature on a topic area” [36], in our case herbal products investigated in clinical trials for caries prevention.

## 2. Materials and Methods

Scoping reviews are particularly helpful in cases where the subject has not yet been comprehensively reviewed or it is heterogeneous [36], and both features are true for the theme of this review. We followed the methodology generally accepted in the field of scoping review (as proposed by Arksey and O’Malley [37] and further developed by Levac et al. [38]), with the five basic steps of the investigation:

- Formulating the research question(s)
- Searching for the published data
- Selecting the relevant studies
- Mapping the data
- Assembling, summarizing, and communicating the results

### 2.1. The Research Question

This review is meant to answer the following question: what is known about the clinical trials performed to evaluate herbal products intended to be used for the prevention of dental caries? At a more detailed level, the following questions are considered:

What are the herbal products that have crossed the non-clinical stage of research and have entered clinical testing?

To what extent have these products been assessed in non-clinical settings (particularly from the point of view of efficacy)?

What are the dosage forms used in the clinical trials performed so far in this field?

What Phase the products evaluated were in as part of the clinical development?

What is the quality level of the studies performed thus far? 

What do we know about the age and gender of the subjects included in those trials? 

What was the duration of the intervention in these trials? 

What were the positive/negative controls used in these trials?

What were the primary endpoints used? 

What are the main results up to now?

A scoping review protocol has been prepared and agreed by those involved in writing this paper and is available through Figshare (https://figshare.com/articles/Clinical_trials_with_herbal_products_for_the_prevention_of_dental_caries_and_their_quality_protocol_for_a_scoping_review/7314338).

### 2.2. Searching for the Published Data 

Taking into account the methodology specific for scoping exercises, which is oriented towards the use of a broad base of literature, we used several literature databases: Medline (through the electronic interface PubMed), Scopus, ISI Web of Knowledge, Lilacs, and Cochrane Reviews; In addition to these sources, we also made searches in three clinical trial registry databases: Cochrane Clinical Trials, EU Clinical Trials Register, CliniclTrials.gov. In order to avoid narrowing the results through complex queries, we preferred using a small number of key-words: “plant AND caries” and “herbal AND caries”; to ensure maximal sensitivity no additional qualifiers referring to the type of study (e.g., “clinical trials” or “prevention”) were used. No date or language restriction was applied. The search process was finalized on 10 October 2018.

### 2.3. Study Selection

We kept the review focused on products that have been subject to clinical investigations. Publications reporting on clinical trials (in humans) for caries prevention were included, irrespective of the age, degree of severity, endpoint, or route of administration. Papers where the objective of the clinical investigation was related to the plaque and gingivitis control were not included, although there is a strong connection between plaque, caries and gingivitis. Instead, papers reporting on the clinical antimicrobial efficacy of mouthwashes in the context of caries prevention were retained. Relevant grey literature (particularly guidelines) were also looked for using appropriate keywords in search engines.

Non-clinical investigations, such as “ex vivo”, “in vitro” (e.g., those performed on standardized bacterial strains or on clinical isolates), “in silico”, “in rodents” or other animal studies were excluded. In situ studies using enamel blocks worn by human volunteers were also excluded as not providing direct evidence on the caries prevention effects of the interventions. We also excluded studies assessing the influence of herbal extracts on the clinical performance of glass ionomers.

Studies focused on fluoride extracted from herbal sources were excluded, because fluoride is expected to have the same therapeutic effects irrespective of its source or production process; studies with fluoride-impregnated miswaks (chewing sticks) were similarly excluded. Antibodies or similar proteins obtained in plants as expression systems (e.g., antibodies manufactured in tobacco plants), animal-derived products (e.g., propolis), breakfast-enriched cereals or other solid foods (e.g., oat hulls), products acting by a non-pharmacological mechanism (e.g., acemannan used in direct pulp capping of primary teeth), and homeopathic products were left out. Studies on less than 8 subjects were considered mini-series or small collections of case reports rather than full clinical trials (irrespective of the way in which the reporting authors labelled them), and consequently they were not included. Non-interventional (observational) studies, either longitudinal or cross-section, reviews, or ethnopharmacology studies were omitted. Reviews, editorials, or opinion articles, while not included, were taken into account in the interpretation of the data and for retrieving potential additional studies. Were we could not get access to the full text of a published abstract, we contacted the correspondence author to solicit a reprint or further information, if an e-mail address was available.

### 2.4. Data Mapping

Paper titles and abstracts returned by the searches were screened by two independent members of the team, and those considered relevant were retrieved full-text and used for data charting. Any divergences among the two main reviewers were solved with the implication of a third member of the team. For each publication the following data were extracted in a data collection form:

- Stated or implicit purpose/objective of the clinical trial
- The dosage form evaluated
- The herbal source on which the dosage form evaluated is based (name of the plant species, part, and additional elements of interest, if any)
- The phase of the clinical development (Phase I, Phase II, etc.) and non-clinical data on efficacy
- The number of centers involved in the trial (single-centric or multi-centric study?)
- The country/countries in which the trial has taken place
- Data on the quality of reporting the trial results and the risk of bias affecting those trials (randomization, concealment of allocation, blinding, control)
- Study design (parallel, cross-over, n-of-one, etc.)
- Demographic info about the patients (age and gender)
- Duration of the intervention
- Primary endpoint
- Main results of the study

For the assessment of bias, the Cochrane Collaboration’s tool for assessing risk of bias (version 5.1.0) [39] was used, with the guidance accompanying it, and appropriate adaptations taking into consideration the different purpose of the scoping review (versus a systematic review and meta-analysis, for which the Cochrane tool is intended). To assess the extent of non-clinical data on efficacy for the products reviewed we performed Medline searches using the name of the plant species and “caries” and manually screening all the results to identify non-clinical efficacy data.

### 2.5. Collating, Summarizing, and Reporting the Results

The data collected were coded and where relevant, compared and analyzed by qualitative and where possible, quantitative means, the narrative synthesis being structured around the main questions of interest of the study, as detailed in “The research question” section.

### 2.6. Consultation

This is an optional step and was performed after the first draft of the paper, by submitting it to one expert in caries prevention, two experts in the field of herbal medicine, and two experts in the field of pharmacology and clinical pharmacy.

## 3. Results and Discussion

**Figure 1:**
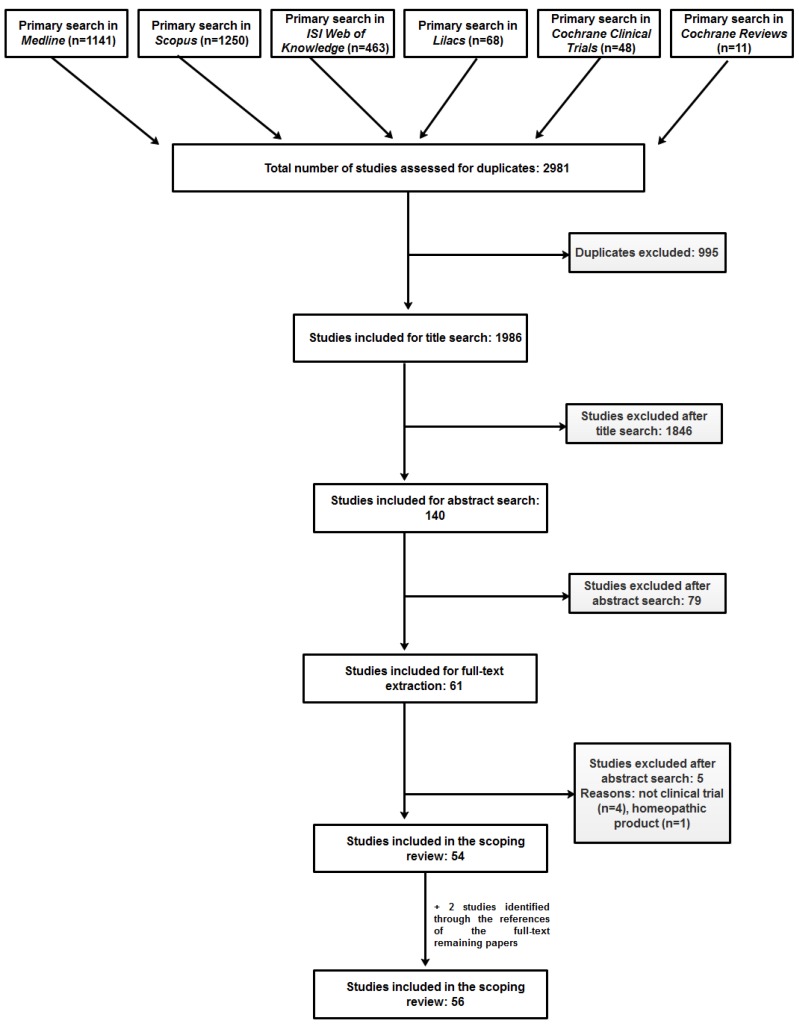
Prisma flow diagram showing the paper selection process.

One thousand nine hundred and eighty-six unique papers were retrieved from the different sources and screened to give a final pool of 56 articles, reporting on the same number of clinical studies (Figure 1). We could not gain access to the full-text of four publications for which the abstract was available (one was only published as a meeting abstract); in one of the four cases we contacted the correspondence author and asked for a reprint, but no reply was received; for the other three cases we could not find an e-mail address of the corresponding author.

### 3.1. Study, Purpose and Objectives

The large majority of studies published include in their introductory section one or a few sentences describing the main purpose or objective of the clinical trial reported on. In most cases, the objective was the investigation or evaluation of the effects of a certain herbal product on bacteria from the oral cavity, assumed to be the main cause of caries. In half of the studies (28/56), in stating the purpose, direct reference was made to the effect on salivary *S. mutans*, for instance “to assess the effect of rinsing with green coffee bean extract in comparison with chlorhexidine mouthwash and sterile water on salivary *Streptococcus mutans* count” [40]. In a smaller number of cases, reference was made to other bacterial or fungal species, most often besides mutans streptococci: lactobacilli (6 papers) and *Candida albicans* (2 articles). Other papers, stating the purpose made general references to the effect on the dental biofilm (n = 2), to cariogenic microorganisms/microflora or microbial counts (n = 9) or to the implicit allusion to an antimicrobial effect by stating the purpose as evaluating certain extracts “as mouthwashes” (n = 2). In other words, in over 83% of the papers included in our review (47 out of 56), the purpose was stated in broader or narrower terms of antimicrobial effects. In a minority of papers, the purpose was formulated with reference to other variables assumed to be relevant for the cariogenic processes: plaque, pH, salivary secretion, or certain ion concentrations (Ca^2+^, PO_4_^3-^) (n = 5) or the effect on human salivary amylase (n = 1). In two cases the objective was explicitly stated as the assessment of the anti-cariogenic effect or caries-prevention effect of the intervention, whereas in a small study the objective was of a purely exploratory nature: to investigate “the effects on oral conditions of adding three oranges per day to the diet of children already receiving a balanced diet”.

Understanding the purpose and objective(s) of the published studies is important because they are (or should be) in a direct connection with the primary endpoint(s) used in designing and carrying out the trial. As shown by this summary, trials have been primarily focused on efficacy and less on safety.

### 3.2. Dosage Forms

**Figure 2:**
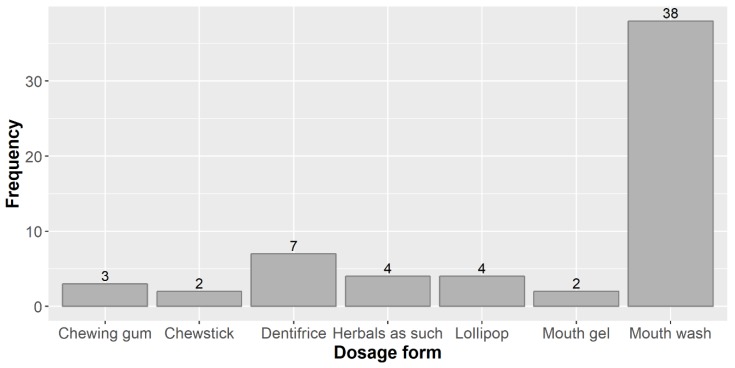
Dosage forms used in the studies reviewed and their frequency.

In the 56 papers reviewed, a number of 60 dosage forms were used, because in two studies, more than one dosage form was investigated (three in one case and two in an additional two studies). The dosage forms used in these studies and frequency of their use are shown in Figure 2.

By far the dosage form most widely used in the studies under review was the mouth wash (MW) or mouth rinse (the two terms are for all practical purposes synonymous, but the official term in the European regulatory system is “mouth wash” [41]). This is in line with what other authors report (without a quantitative backing), e.g., “For many years, MW has been the most frequently tested vehicle for antimicrobial compounds” [42]. A critical review on how herbal products contributed to oral care (not exclusively focused on caries preventive herbals) also found similar results: 47.5% of the products included in that study were MWs, whereas toothpastes represented only 7.3% and oral gels 6.0% [43].

What is the reason for using MWs so extensively? Does this dosage form certain clinical advantages over the others or does it just happens to be more convenient (easier to prepare and possibly to administer)? It has been argued that mechanical elimination of the bacterial plaque through brushing and flossing performed regularly and in an effective manner is the key solution to lowering the risk of caries development, and MWs should be used only when in conjunction with these mechanical means [44]. It would seem therefore that using herbal extracts or other herbal products for oral hygiene in dentifrices is a more logical approach for increased efficiency, but dentifrices were noticeably less frequent than MWs. One may also question the residence time of the active ingredients in the mouth when using a simple MW, likely to be washed out by the salivary flow. In the case of chlorhexidine gluconate at least, it has been demonstrated that significant amounts of the antiseptic substance are detected up to 12 h after the last application of a MW, which indicates that this should not be a concern [45]. Certainty is not to be had, though, in the case of the different herbal products, because it is known that other compounds than chlorhexidine, such as several quaternary ammonium salts have shown good efficacy in vitro, but little or no plaque inhibitory action in vivo, a fact likely related to the residence time and delivery characteristics in the oral cavity of these potential antibacterial compounds [46]. Throughout the clinical studies performed up to date we have identified generally no interest for estimating the residence of the active ingredients in the oral cavity or other aspects of the topical delivery of the active ingredients.

Mouth gels have higher viscosity, attained by use of a variety of bioadhesive polymers, and therefore they may ensure a longer residence time of the active ingredient(s) in the oral cavity in comparison with MWs, resulting thus (in theory) in better efficacy. Additionally, this longer residence may lead to less frequent administrations and lower amounts of active ingredients, improving patient acceptance and adherence to treatment [47]. The biopharmaceutical considerations for compounds acting in the mouth are more complex and not limited to the residence time; but if the active ingredient is not adsorbed or bound within the oral cavity, it will have to exert its pharmacodynamics effects in a time interval shorter than 15 min, a rapidity of action that few compounds have [46]. Direct (head-to-head) comparisons in similar conditions of use for MWs and gels are limited in the literature. A systematic review, based on a small number of relatively heterogenous studies found that in direct comparisons, chlorhexidine MWs had better efficacy (but also additional safety issues) than dentifrices and gels in three out of five studies [48]. However, that review grouped together gels and dentifrices, and if the latter are excluded, the results become inconclusive (in one study the gel had better results than MW, in another a gel formulation was equally effective as a MW, whereas a dentifrice gel was inferior (but this should rather be considered a dentifrice than a gel). Moreover, this review was limited to chlorhexidine, which is known to be sensitive to interactions with some of the ingredients of the dentifrices such as anionic compounds, abrasives, calcium, and sodium monofluorophosphate, reducing its availability and/or activity. In the case of fluoride the interest for gels seems to have decreased in the favor of fluoride varnishes [49], which ensure even longer residence times of the active ingredient (fluoride) in the mouth. It seems quite unreasonable to extrapolate from this meager prior knowledge on chlorhexidine to herbal ingredients with different chemical and biological properties. One study reviewed here compared three formulations of *Lippia sidoides* Cham. essential oil: MW, mouth gel, and dentifrice; only the dentifrice had a statistically significant effect in terms of colony forming units of *S. mutans* [50]. The question remains therefore open whether MWs are the ideal dosage form or if dentifrices and gels could offer better alternatives for local delivery of caries protection agents.

A sugar-free lollipop based on a licorice extract was investigated in four clinical trials, performed in different parts of the world [51,52,53]. This is not a conventional dosage form, but rather one migrated from the field of confectionary to that of health. It has been argued that it can be used for oral health purposes across a broad spectrum of patient ages, and that such herbal lollipops should be recommended as an alternative to conventional, cariogenic lollipops [54]. Blurring the line between traditional sweets and medicines or medicines-like products might still rather encourage the use of the former in the detriment of health or the excessive use of the herbal lollipop, perceived as risk-free, with unwanted consequences. Concerns over the potential abuse of such lollipops and the need of the dental practitioners to educate patients to stick to the recommended doses have already been expressed in the medical literature, particularly because glycyrrhizin, the main ingredient of the licorice extract is apt to induce pseudoaldosteronism by inhibiting 11β-hydroxysteroid dehydrogenase type 2, the enzyme involved in converting cortisol to the less active cortisone [55].

Chewing gum has tended to bear a transmutation from a mere pleasurable item to a health-promoting product, a phenomenon that paralleled the substitution of sugar as a sweetener with polyols, particularly xylitol [56]. Whereas xylitol- and other polyol-sweetened chewing gums have a potential of reducing the risk of caries development by increasing bicarbonate-rich salivary flow and a direct effect of polyols against microbial organisms through the creation of a net energy loss (“the futile cycle”) [56], chewing gums become also increasingly attractive as dosage forms of their own, for a variety of active ingredients intended to act topically (in the mouth) or systemically [57]. The use of the chewing gum as a dosage form for herbal ingredients in three of the studies reviewed here is in this context not surprising.

In four studies the investigated herbal products assumed to have caries preventive actions have been used as such, with no pre-processing or extraction: these were mostly seeds, small fruits containing seeds (achenes, e.g., *Foeniculum vulgare* Mill.) or a fresh sliced orange taken at the end of each meal, and in a single case, fresh leaves (*Ocimum tenuiflorum* L.). Chewsticks (*Garcinia mannii* Oliv.) or miswack sticks (*Salvadora persica* L.), employed in two studies, are also as a matter of fact unprocessed or minimally processed herbal products.

### 3.3. Herbal Products Evaluated in Clinical Trials

The majority of cases, the herbal products evaluated in the trials reviewed here were derived from a single plant species, but in a smaller number of cases, they were more complex formulations, obtained from three or more distinct species. A number of 67 species have been used in the 56 sources reviewed here, of which 31 have been clinically evaluated singly, whereas 36 additional species were only part of complex products used and assessed for caries prevention (Table 1). The families most represented were Asteraceae, Fabaceae and Lamiaceae (5 species each), Myrtaceae (4 species), Apiaceae and Rutaceae (3 species each), Combretaceae, Ericaceae, Lauraceae, Rosaceae, Rubiaceae, and Zingiberaceae (2 species each). Among the species investigated singly, most studied was *Camellia sinensis* (L.) Kuntze (leaf), with a number of seven trials, *Terminalia chebula* Retz. (fruit), evaluated as monotherapy in 5 trials and in combination with other products in four additional trials, and *Glycyrrhiza uralensis* Fisch. (root), looked over in 6 trials as monotherapy and in an additional one in combinations. Unfortunately, in many cases the authors of the reporting papers did not provide minimal details on the herbal source besides the name of the plant species: for 37 out of the 67 species, the part used (e.g., root, leaf, flower, fruit, seed, etc.) was not stated, whereas in the case of extracts, with minimal exceptions a full characterization was lacking (solvent, drug extract ratio, D.E.R., compound(s) used for standardization purposes, etc.). In one case, although the authors used repeatedly throughout the paper the term “extracts”, it seems that they only tested pure stevioside and rebaudioside A, and not extracts proper. In most cases the products seem to have been used as fluid or dry extracts (mostly aqueous or hydro-alcoholic), but essential oils, fatty oils, a high molecular nondialyzable material obtained from concentrated juice, a gum (mastic), or fresh juice, were also used. In a small number of cases seeds, achenes or leaves were used as such, with no additional preparation.

### 3.4. Clinical Study Phase and Non-Clinical Data on Efficacy

Clinical trials of new medicinal products are generally carried out in a sequential manner, in steps known as “phases”. Although criteria, methodological considerations, and definitions for each phase are not univocally agreed upon, it is widely accepted that clinical classified in one of four possible phases (from phase I to phase IV, sometimes with more granularity, e.g., phase IIa and phase IIb), each being intended to answer different scientific questions [58]. For instance, phase I trials are the first clinical evaluations in a formal framework in humans, include small numbers of subjects and are basically focused on safety aspects, whereas phase III trials are usually large (including a high number of subjects) and are often called pivotal, because the data generated in them will be used to make significant (regulatory) claims about the product evaluated [59]. We assessed the sources collected for review in order to have an understanding of the clinical stage of development and the maturity of the clinical research with herbal products for caries prevention. However, the curious finding of our review was that in every single case the authors did not use the classical terminology of “phases”. Only in four cases the trials were described as “pilot” (which one might be tempted to interpret as “phase I”, but such a label may not be necessarily correct), and in a fifth one the authors stated that the reported trial was “preceded by a pilot study of 7 days using 10 volunteers, students of dentistry”, but no phase label was affixed to the main study reported there [60]. This aspect could be explained by the fact that the scientific community operating with this type of research is not familiar with the phase classification of the clinical development. This is, however, rather unlikely; it seems rather more likely that the relevant scientific community is familiar with the terminology and classification, but considers it inappropriate for clinical context of herbal products used for caries prevention (e.g., as being too “atypical” from the majority of clinical trials). This may also be related to the fact that in the case of conventional medicines, clinical trials are most often sponsored by companies with sufficient financial resources, who will use the results for regulatory submission purposes, and since the “phase” language of the clinical trials is part of the pharmaceutical regulatory jargon, they are careful to have such a description included in the trial protocol and the study reports, whereas in the case of our review not only is the field atypical (caries prevention), but such studies are most often sponsored by small companies or the academic environment, lacking both the interest and attention to such details. In any case, it would be useful, if not necessary, to have a discussion on the adoption (or reasons of not adopting) the conventional terminology of clinical phases for trials investigating herbal products in caries prevention.

A synthesis of the non-clinical data available for the herbal species included in this review is shown in Table S1; we limited our searches to those species investigated as single therapy in clinical trials, excluding the combinations of multiple species. For about one third of the 31 species (35.48%) we could find no non-clinical efficacy data; for two thirds of the species at least one or several in vitro data were available, the large majority showing anti-microbial effects against oral pathogens, particularly *S. mutans*. Only for two species (*Camellia sinensis* (L.) Kuntze and *Psidium cattleianum* Afzel. ex Sabine) we could identify in vivo non-clinical data, consisting of caries models in rats, in whom caries were induced by *S. mutans* infection and feeding a cariogenic diet [61,62,63,64]; three of these four rat studies were carried out in the 1990s, suggesting that more recently such rodent efficacy studies have not been considered necessary or relevant, the investigation proceeding for most herbal products from the in vitro data directly to the clinical testing. This is coherent with a number of review papers that challenged the usefulness of animal experiments for the development of new medicines or clinical interventions, although the limitations could be attributed to imperfect design, performance and reporting on animal studies and on this issue the jury is still out [65,66]. Since in the European Union toothpastes and mouth rinses are regulated as cosmetic products, there is no regulatory guidance on the non-clinical and clinical development of caries prevention products. In the United States FDA has a certain authority and control over oral care containing fluoride, but it has been estimated that the evolution of the oral care products there has rather been driven by marketing interests than the public best interests [65]. In that regulatory framework an interest has been manifested for the reduction of animal caries testing, with several alternatives proposed, but those are limited to fluoride products [67,68]. For non-fluoride products such as herbal dentifrices or mouth rinses, there is currently no regulatory guidance on the need for animal studies and the studies reviewed here indicates that in the majority of cases the clinical testing has been preceded by (more or less limited) in vitro studies only.

### 3.5. Monocentric vs. Multicentric Studies

Clinical trials may be performed in a single center (i.e., they are monocentric) or simultaneously in multiple centers (multicentric trials). Although challenging from the perspective of running with sufficient homogeneity and cohesion, the latter have obvious advantages with respect to the confidence in the results obtained and their generalizability. These advantages are related to the fact that more centers allow an appropriate sample size of subjects, a more rigorous assessment of efficacy and safety (if the results are consistent among different centers), higher diversity of subjects, and less selection bias [69]. None of the trials reported by the sources included in our review was multicentric. This implies a higher risk of bias and a lower generalizability of results.

### 3.6. Geographic Origin of Studies

We were interested to have a quick overview of the countries and regions involved in performing the clinical trials included in our scoping review, so as to detect whether the interest for the topic is limited to certain regions of the world or wider, and what are the countries where such studies are carried out.

**Figure 3:**
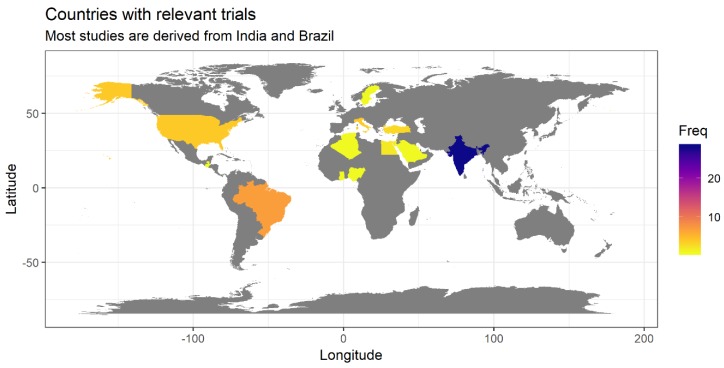
Geographic origin of studies reviewed in this paper (frequency is shown in absolute numbers).

All the sources reviewed were derived from trials performed in 14 different countries of the world. Half of the sources included in the review (28 out of 56) were based on clinical studies performed in a single country: India. Other countries where multiple studies of interest for this review were performed were Brazil (7 studies), Italy, USA (4 studies each), Turkey (3 studies), and Egypt (2 studies) (Figure 3). The analysis by continents indicated that studies carried out in Asia were by far the most numerous (34 out of 56), covering more than half of the whole body of research. It is interesting to note that although China is usually one of the large centers of clinical research in the field of herbal therapy, we identified no study of interest for this review (caries prevention) performed in this country.

### 3.7. Study Design

By far the most widely design employed was the parallel one, three quarters of the studies inventoried here making use of this design (41/56, 73.21%; an additional study seems also to have used a parallel design, but the text did not allow a firm conclusion in this sense). Six additional studies (10.71%) used a cross-over design, and eight (14.29%) used a before-after (pre-post) design. Each of these designs have their own advantages and shortcomings. The parallel design is the most widely used and the most apt to provide convincing evidence of efficacy and safety, if sufficiently powered. Parallel design trials are also apt to be multi-centric, which despite logistic difficulties, assure broader generalizability of their findings, whereas cross-over trials are monocentric [70]. It has been suggested that parallel designs are more appropriate for pivotal trials, whereas cross-over trials are more useful for proof-of-concept purposes [70]. Pre-post trials are particularly susceptible to a number of biases and, although the subjects theoretically serve as their own controls, most often a separate control group is not used; therefore such studies should be actually labelled as “uncontrolled” [71].

### 3.8. Patient Demographics

**Figure 4:**
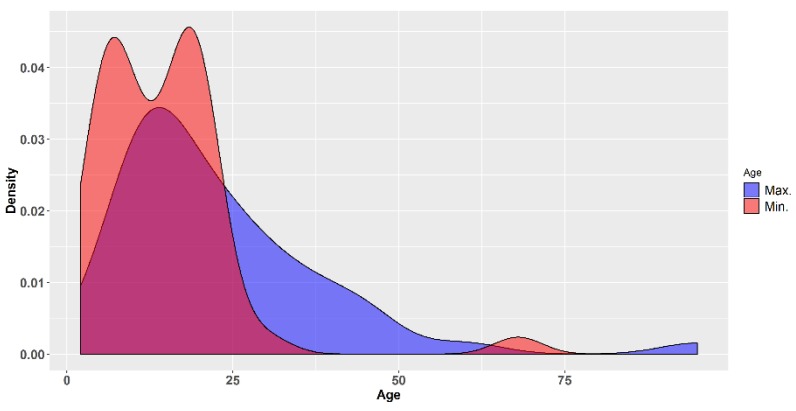
Density plots of the minimum and maximum ages of the subjects included in the clinical trials reviewed.

The largest majority of studies included young and very young subjects. For instance, of the 40 studies for which the maximum age of the participants was stated, it was larger than 35 years in only seven studies (17.5%) and only two studies (5.0%) included subjects older than 50 years of age. In 90% of the subjects for which the maximum age was reported, it was lower than 35 years (Figure 4). This means the most of the subjects in which these studies were performed were young and very young (half of the 40 studies in which the maximum age was reported were less than 18 years of age). From a certain point of view this may seem encouraging, because children have often been neglected and not included in the development of new medicines, leading the authorities in different parts of the world to adopt legislative measures designed to stimulate the clinical research of medicines in the paediatric population [72]. On the other side, it is rather worrying that most products are investigated only in children, in the absence of safety data from adults. Moreover, it is not clear to what extent data generated in such young subsets may be relevant for the use of those herbal products in the adult and geriatric population. This brings us back to the fact that the greater need is in the latter subpopulation: “there is a lack of evidence for caries preventive methods in adults with increased caries risk” [21,24]. It is also regrettable that in a number of trials the age of the participants was not reported, or reported only in general terms, such as "adult volunteers" or “different ages”. The demographics (such as age and gender) of the subjects included in a trial is not a mere minor detail, but an important feature of the sample studied and should always be reported accurately (for instance they are part of the basic information required by legislation in the USA for the trials registered in the ClinicalTrials.gov database) [73].

In almost two thirds (33 studies, 61.11%) of the studies reviewed the exact gender distribution of the subjects was not reported. In those 21 studies where gender distribution was reported the total number of male subjects was slightly higher than that of the females (675 vs. 531). In many studies no information was provided on the proportion of the two genders, whereas in other studies general statements such as "both sexes were included” or “both genders” were considered sufficient. This is obviously a flawed way of reporting that needs improvement.

### 3.9. Duration of the Intervention

Over 60% of the 56 studies reviewed by us had a duration of the intervention (i.e., of administering the herbal products evaluated) of less than one week and one third of the studies (20 out of 56) evaluated the herbal products following a single application (one day). The value of such very short duration studies may only be exploratory and hypothesis generating. It has been recognized that current standards should be trials of 2–3 years length, but in the same time the need (for pragmatic reasons) to develop methodologies able to allow shorter durations has been acknowledged [74]. The problem with the short duration trials is that even if an effect is shown, uncertainty will remain about the ability of that specific product to maintain the preventive effect on a long time. Therefore, long-term studies will likely continue to be needed, even if improved methodologies for showing short time benefits are developed.

### 3.10. Control Choice

In almost one third of the studies reviewed (n = 17, 30.36%) chlorhexidine in different concentrations and dosage forms was used as a positive control. In 10 studies (17.86%), mostly those with a pre-post design, no control was used (the authors assuming that in such cases the patients served as their own controls, an invalid assumption, as mentioned above). Water, saline solutions, other placebo options (e.g., mouthwash or dentifrice with no active ingredient) or passive controls (i.e., no intervention) were used in a number of 22 studies (39.29%). Other controls used, depending on the dosage form and herbal product investigated included xylitol, fluoride mouthrinse, a thymol/carvacrol mixture (mouth rinse and mouth gel), sucrose, a “regularly available low abrasive dentifrice” (commercial name not provided, but manufacturer indicated in the published paper), commercial mouthwashes such as Plax and Listerine, commercial toothpastes (Colgate), and ACP-CPP chewing gum. Whereas some of these active controls are reasonable, the justification for a minority seems less convincing (for instance, a thymol/carvacrol mixture was used as a control because the herbal product assessed contained the two phenols).

In a non-negligible number of studies (n = 10, 17.86%) two or even more controls were used, for instance a positive control (e.g., chlorhexidine) and a negative control (distilled water, saline, placebo, etc.). This may be a more than reasonable approach, but because the sample sizes were in almost all cases very low, including two or more control arms diluted even more the statistical power of those studies. Including both a negative and a positive control should be performed when a trial has low assay sensitivity, but if there is likelihood of assay sensitivity, particularly when non-inferiority towards the active control is claimed [75,76], a larger sample size is needed, which the overwhelming majority of trials reviewed by us did not provide. Moreover, in a case both a “herbal” and “synthetic” dentifrices were used as active controls; this is in theory apt to provide insight about the effectiveness of the tested product in relationship with other herbal product toothpaste or with a “synthetic” one, but in the absence of sufficient statistical power, it actually provides concluding evidence for none.

### 3.11. Primary Endpoints

A primary endpoint is the outcome used in a clinical trial to evaluate whether an intervention is effective [77]. According to the ICH guidelines on Statistical Principles for Clinical Trials, “The primary variable (‘target’ variable, primary endpoint) should be the variable capable of providing the most clinically relevant and convincing evidence directly related to the primary objective of the trial. There should generally be only one primary variable” [78]. None of the studies reviewed here used the term “primary endpoint” and none described one. Some studies used a single endpoint, some multiple endpoints, but none qualified one of the endpoints as “primary”.

44 out of 56 studies (78.57%) used microbial counts (usually as colony forming units (CFU), but other means were also used in fewer cases) as one of the endpoints, often the only one. Such an endpoint is a surrogate one, because it does not measure directly the effect on caries; it measures the impact on microbial counts, with the assumption that a decrease in microbial counts should translate into a decrease in caries incidence or severity. “History has taught us to be cautious about the use of surrogates. We can be led astray too easily” [79], because although a surrogate marker may decrease, the impact over the main objective may not be the one expected or to the same extent as expected from the measurement of the surrogate and in different clinical areas examples are not lacking [80,81]. Thus, such an endpoint if confirmed in a clinical trial may provide evidence that an intervention (a herbal product) might be worth exploring for its potential caries preventive effect, but it does not provide direct evidence in favor of such an effect.

Other endpoints were used considerably less (only once or several times) in the studies reviewed here: the Quigley-Hein plaque index (as modified by Turesky), OHI-S index (Simplified Oral Hygiene Index), the gingival bleeding index, or a similar gingival score (the Loe–Silness gingivitis index), a calculus score, caries activity (measured by Oratest), the plaque or salivary pH, salivary buffering capacity, salivary flow rate, salivary amylase activity, calcium and phosphorus concentrations, and DMFS (decayed, missing, filled, applied to tooth surfaces) score. Each of these is more or less remotely correlated with the caries prevention activity, being thus surrogate or soft endpoints. DMFS is the only one that measures directly the caries status, but due to its nature, it is likely not to be a sufficiently sensitive metric when used in short duration interventions. Since most studies included in this review were of short duration, this is probably the reason for which DMFS was used in one study only [82].

### 3.12. Quality of Reporting and Risk of Bias

Although the Cochrane Collaboration’s tool for assessing risk of bias (version 5.1.0), considering the purpose of systematic reviews, recommends focusing on the risk of bias and not the quality of reporting, it acknowledges that the quality of reporting is important and may impact the ability of both authors and readers to judge the dangers of bias; its shift of emphasis from the quality of reporting to the risk of bias is motivated by the fact that the risk of bias is not straightforwardly related to the quality of reporting. For the purpose of this review, we considered that evaluating the quality of reporting is equally important, because understanding the limitations and issues of current reporting practices offers to the scientific community an opportunity to improve reporting of future research.

**Figure 5:**
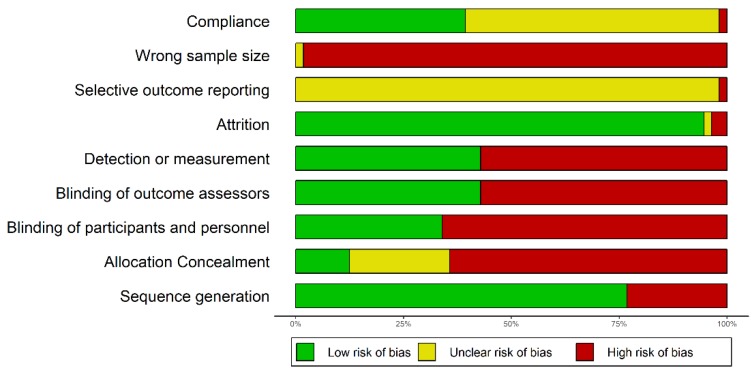
Graphical representation of the risks of bias in the trials reviewed.

All trials reviewed here were ascertained to be of overall weak methodological quality (Figure 5). About one quarter of the trials did not use randomization at all, which opens the door for selection bias. Although over 75% of trials were randomized, in most cases the reporting was limited to stating this fact, but no additional information on the randomization process was provided. In the few cases where randomization was described in slightly more details it was not clear enough to understand the process (or type of process), except for references to a “lottery method”. Concealment of allocation was ensured in seven trials (about 13% of all trials), all of them using a lottery method for randomization (in the risk of bias graph, the zone with “unclear risk” for concealment of allocation corresponds actually to the non-randomized studies, for which concealment of allocation makes no sense). In only one third of the trials participants and personnel were blinded, whereas the large majority of the studies were opened for both patients and those administering the intervention. It has been shown that “double-blind” is a phrase susceptible of multiple interpretations for the healthcare professionals, whereas the term “single blind” is “unhelpful without clarification” [83]. One of the studies reviewed was described just like that: “single blind” with no additional clarification. In both single and double blind trials, of key importance is whether or not the examiner (outcome assessor) is aware of the intervention whose results are assessed [83]. It is encouraging that the proportion of studies where the examiner was blinded was higher (although not with a large margin) than the proportion of studies were neither patients nor the personnel were blinded (42.59% vs. 33.33%).

Attrition bias was generally of no concern for the large majority of studies reviewed here (it affected only two trials and uncertain for a third one, for which we could not get access to the full text). However, this strength of the studies seems to be directly related to another weakness that will be discussed separately: the short duration of most studies.

Whether or not the reporting was selective could not generally be assessed, because for no study the primary and secondary endpoints were clearly stated in the published paper, to allow an uninformed reader to understand to what extent the reporting was complete or not.

**Figure 6:**
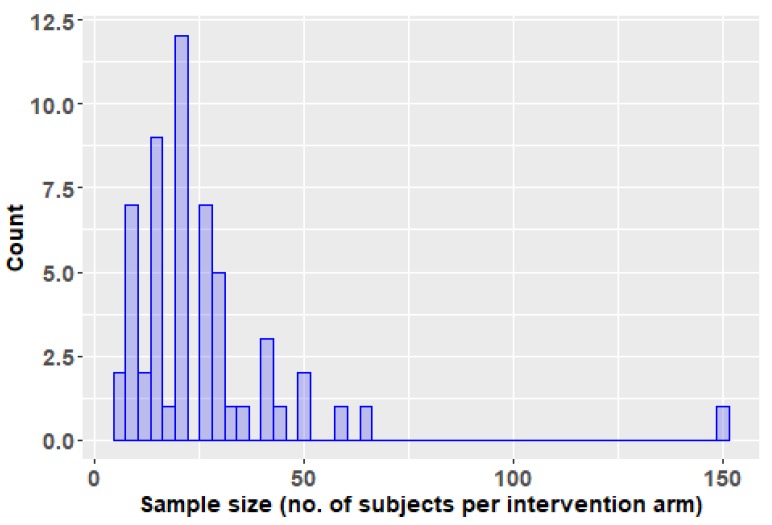
Histogram of the number of subjects per intervention arm in the trials reviewed in this paper.

All studies reviewed here for which we had access to the full text could be judged as affected by a wrong sample size bias. Among the 56 trials evaluated by us, only one had more than 100 subjects per arm of treatment and only three trials had more than 50 subjects per arm of intervention. More than half of the studies (33 out of 56) included 20 subjects of less per arm of treatment (Figure 6). The small sample size leads to multiple problems of reliability, some related to different biases, and some manifest even in the absence of other sources of bias, including an overestimation of effect if the result is not a mere chance finding (“the winner’s curse”) [84]. Such studies with herbal products, cannot, therefore, be used to base clinical decisions on them, but only for exploratory purposes. In the case of a chlorhexidine dental coating evaluation for its caries prevention effect, the initial studies included total numbers of 240 and 1240 patients, whereas the pivotal trial was designed with a sample size of 1000 subjects, intended to allow the detection of a 20% difference in net caries increment [85]. This is the kind of study with sufficient power to allow basing decisions for clinical practice. Moreover, as it has been shown in the relatively recent literature, because currently it is unethical not to provide all subjects in such a trial to fluoride, a different product (such as a herbal one) will have to provide a beneficial effect against besides that of the fluoride, and thus larger sample sizes are required [74].

The compliance bias risk was estimated to be low in about 37% of the studies reviewed, either because the authors assessed compliance or because the study intervention was limited to one day and it could be concluded that the risk of non-compliance was low. In about two thirds of the studies compliance was not assessed, and in a single trial the authors assessed it and discovered that the adherence decreased from an initial 78% in the first three days to around 60% in the rest of the day (with an excursion down to 54% in days 7–14) [52].

### 3.13. Clinical Trial Results

85.71% of the 56 studies reviewed reported positive results, at least for some of the endpoints used, if not for all. Some of the 14.29% of the studies that had negative results based on the conventional significance level 0.05 tended to present the results in a positive light. For instance, one study that found no significant difference among four groups (*p* = 0.602), of which two had received active controls, one the test product and one a placebo product concluded that “*B. dracunculifolia* had the same efficiency of the materials used to oral hygiene in reduction of dental plaque and, consequently, prevention of dental caries” [86]. A more likely conclusion in this case would be that the study did not have sufficient power to discriminate between the active interventions and placebo. Two other studies [46,47] found that only the high-risk subgroup (children) reached the endpoint (had a significant decrease in the *S. mutans*). Although this could be a real effect, it seems equally likely (if not more) than it might be simply regression to the mean, because groups with highest risks are most likely to be impacted by this now classic artifact [87].

## 4. Conclusions

A number of no less than 56 clinical trials have been performed assessing the potential use of herbal products in caries prevention. Most of them were focused on assessing the antimicrobial effects of the products tested, primarily on *S. mutans* and to a lesser extent on other microbial or fungal species. In a minority of cases other variables were of interest, such as plaque, pH, salivary secretion, ion concentrations (Ca^2+^, PO_4_^3-^) or the effect on human salivary amylase. Mouthwash was the most widely used dosage form, whereas dentifrices and other dosage forms were much less employed. In the largest share of the studies the herbal products evaluated were derived from a single plant species, whereas in a smaller number of trials complex formulations obtained from three or more distinct species were used. 67 species have been investigated thus far, of which 31 alone and 36 as part of complex products. In none of the trials reviewed used the authors the classical terminology of “phases”; only in four cases the trials were described as “pilot”. None of the trials reported by the sources included in our review was multicentric. All trials reviewed here were ascertained to be of overall weak methodological quality. The large majority of studies used a parallel design and included young and very young subjects. Over 60% of the studies had a duration of the intervention of less than one week and one third of the studies they evaluated the herbal products following a single application (one day). In almost one third of the studies reviewed chlorhexidine was used as a positive control, whereas in 17.86%, mostly those with a pre-post design, no control was used. Four out of five studies used microbial counts (usually as CFUs) as one of the endpoints, often the only one. 85.71% of the trials reviewed reported positive results, at least for some of the endpoints used, if not for all, but given the methodological weakness and biases affecting them, it is hard to conclude on the efficacy based on the studies conducted thus far.

## Figures and Tables

**Table 1:** Plant species investigated in clinical trials for caries preventive effects.

No.	Species	Family	Plant Part Used	Product	Frequency	Reference
**Species Investigated as Single Therapy**						
1.	*Coffea canephora* Pierre ex A.Froehner (reported as “Coffea Robusta”)	Rubiaceae	Seed	2% Green coffee bean extract (S Therapeutics, An ISO: 9001-2008 and WHO GMP Certified Co.)	1	M. Yadav et al., 2017
2.	*Baccharis dracunculifolia* DC.	Asteraceae	Not stated. Probably aerial parts or leaf	Essential oil; hydroethanolic extract (D.E.R. not provided)	1	V. Pedrazzi et al., 2015
3.	*Terminalia chebula* Retz.	Combretaceae	Fruit (dried, ground) (with or without seeds)	Dry extract obtained with water, suspended in polyethylene glycol (20% *v*/*v*) and then diluted to 10% with water to get a mouth rinse Dry extract obtained with ethanol 70%, formulated as mouth rinse 2.5% Dry extract obtained with water, suspended in polyethylene glycol (20% *v*/*v*) and then diluted to 20% with water to get a mouth rinse Dry extract obtained with water, suspended in polyethylene glycol (30% *v*/*v*) and then diluted to 10% with water to get a mouth rinse “10% extract” obtained with “normal saline”. (The preparation procedure would suggest that the concentration was 5%–50 g of powder to 1L of saline) Dried 10% (*w*/*v*) extract obtained with distilled water	6 + 4 as combination (*Triphala* *, *Herboral* *^♠^*)	Carounanidy U et al., 2007 Shah S et al., 2018 Nayak SS et al., 2012 Velmurugan A et al., 2013 Palit M et al., 2016 Rekha V. et al., 2014 S. Saxena et al., 2017 Srinagesh J et al., 2012 Srinagesh J et al., 2011 Mishra R et al, 2016
4.	*Terminalia bellirica* (Gaertn.) Roxb.	Combretaceae	Fruit (dried, ground)	“10% extract” obtained with “normal saline”. (The preparation procedure would suggest that the concentration was 5%–50 g of powder to 1L of saline)	1 + 4 combinations (Triphala *, Hiora ^§,^ *Herboral* *^♠^*)	S. Saxena et al., 2017 Srinagesh J et al., 2012 Srinagesh J et al., 2011 Sharma A et al., 2018 Mishra R et al, 2016
5.	*Phyllanthus emblica* L. syn. *Emblica officinalis* Gaertn.	Phyllanthaceae	Fruit (dried, ground)	“10% extract” obtained with “normal saline”. (The preparation procedure would suggest that the concentration was 5%–50 g of powder to 1L of saline) Dry extract obtained with water, suspended in polyethylene glycol (20% *v*/*v*) and then diluted to 20% with water to get a mouth rinse	2 + 3 combinations (Triphala *, *Herboral* *^♠^*)	S. Saxena et al., 2017 Srinagesh J et al., 2012 Srinagesh J et al., 2011 Velmurugan A et al., 2013 Mishra R et al., 2016
6.	*Acacia nilotica* (L.) Delile syn. *Acacia arabica* (Lam.) Willd.	Fabaceae	NA	(Bark, apparently) extract formulated as a toothpaste	2	Patel K et al., 2018 Gupta D, Gupta RK, 2015 [Abstract] **
7.	*Lippia sidoides* Cham.	Verbenaceae	Leaf	Essential oil (used in three formulations: either a 1.4% toothpaste, 1.4% gel, or 0.8% mouthwash in one study, and in two formulations in another: rinse (0.6%, 0.8%, 1%, and 1.2% concentrations) and gel (0.8%, 1%, 1.2% and 1.4% concentrations)), respectively	2	Lobo PL et al., 2014 Lobo PL et al., 2011
8.	*Stevia rebaudiana* (Bertoni) Bertoni	Asteraceae	Leaf	Although the authors of one paper used repeatedly throughout the paper the term “extracts”, it seems that they only tested pure stevioside and rebaudioside A In the second paper the authors made reference to a mouthrinse containing 10% stevia, they probably referred to the sweetener glycosides	2	Brambilla E et al., 2014 Vandana K et al., 2017
9.	*Glycyrrhiza uralensis* Fisch.	Fabaceae	Root	Aqueous and ethylic alcohol extracts (not further characterized) – in most cases further formulated as a lollipop	5 + 1 combination	Peters MC et al., 2010 Hu CH et al., 2011 Jain E et al., 2013 Shah S. et al., 2018 Mentes JC et al., 2012
10.	*Bertholletia excelsa* Bonpl.	Lecythidaceae	Seed	Seed oil (added to a dentifrice)	1	Filogônio Cde F et al., 2011.
11.	*Magnolia officinalis* Rehder & E.H.Wilson	Magnoliaceae	Bark	Bark extract formulated as a chewing gum containing 0.17% extract (magnolol 0.10% and honokiol 0.07%, respectively)	1	Campus G. et al., 2011
12.	*Camellia sinensis* (L.) Kuntze	Theaceae	Leaf	Various extracts, infusions or decoctions, in different concentrations, employed as mouth rinses (e.g., for each rinsing, 1.6 g of leaf powder were suspended for 3 min in 40 mL of boiling water, after which it was kept at room temperature)	7	Ferrazzano GF et al., 2011 Awadalla HI et al., 2011a Awadalla HI et al., 2011b Hegde RJ, Kamath S, 2011 Esimone CO et al., 2001 Thomas A et al., 2016 Thomas A et al., 2017
13.	*Salvadora persica* L.	Salvadoraceae	Sticks (usually not specified whether from roots or branches)	Aqueous extract (e.g., 20% *w*/*w* or 50% *w*/*w* or *w*/*v*) used as a mouth rinse	4 + 1 in combination	Sofrata A et al., 2007 Almas et al., 2004 Chelli-Chentouf N et al., 2012 Jauhari D et al., 2015 Sharma A et al., 2018
14.	*Prunus dulcis* (Mill.) D.A.Webb	Rosaceae	Seed	Seed oil formulated as a dentifrice	1	Aguiar AA et al., 2004.
15.	*Garcinia mannii* Oliv.	Clusiaceae	“Stick” (twig? root?)	Chewing sticks used as such (chewed)	1	Addai FK et al., 2002
16	*Citrus sinensis* (L.) Osbeck	Rutaceae	Fruit	Used as such	1	Dilley GJ et al., 1977
17	*Scutellaria baicalensis* Georgi	Lamiaceae	Root	Extract formulated as mouthwash	1	Kim Y-R et al., 2017
18	*Psidium cattleianum* Afzel. ex Sabine	Myrtaceae	Leaf	Aqueous extract obtained by decoction (16.67% *w*/*v*)	1	Brighenti FL et al., 2012
19	*Vaccinium macrocarpon* Aiton	Ericaceae	Fruit	A high molecular nondialyzable material obtained from concentrated juice (molecular mass cut-off point of 12000), formulated as a mouth rinse	2	Gupta A et al., 2015 Weiss EI et al., 2004
20	*Allium sativum* L.	Amaryllidaceae	Bulb	Extract	2	Chavan SD et al., 2010 *** Thomas A et al., 2017
21	*Foeniculum vulgare* Mill.	Apiaceae	“Seed” (probably achene)	Seed (achene) used as such (1.0–1.3 g)	3	Sultan S, 2016 Swathi V et al., 2016 Ravi VS et al., 2010
22	*Sesamum indicum* L.	Pedaliaceae	Seed	Seed used as such (1 g)	1	Sultan S, 2016
23	*Cocos nucifera* L.	Arecaceae	Seed	Seed used as such (1 g)	1	Sultan S, 2016
24	*Ocimum tenuiflorum* L. (reported as *Ocimum sanctum* L.)	Lamiaceae	Leaf	Fresh leaf used as such (5 leaves)	1	Sultan S, 2016
25	*Plantago lanceolata* L.	Plantaginaceae	Leaf and flower	Infusion formulated as a mouth rinse	1	Ferrazzano GF et al., 2015
26	*Pistacia lentiscus* L.	Anacardiaceae	Mastic gum (resin secreted by the stem)	Gum formulated as chewing gum	2	Aksoy A et al., 2006 Aksoy A et al., 2007
27	*Elettaria cardamomum* (L.) Maton	Zingiberaceae	Seed	Used as such or formulated as a herbal mouthwash	1 + 1 in combination	Swathi V et al., 2016 Sharma A et al., 2018
28	*Eugenia uniflora* L.	Myrtaceae	Fruit (ripe)	Hydro-alcoholic extract, formulated as a dentifrice	1	Jovito V de C, 2009
29	*Dysphania ambrosioides* (L.) Mosyakin & Clemants (reported as *Chenopodium ambrosioides* L.)	Amaranthaceae	Leaf	2% infusion used as a mouth wash	1	Fernandez DKT, 2000
30	*Citrus sp.* (reported only as “lime”)	Rutaceae	Fruit	Fresh fruit juice, formulated with excipients as a mouth rinse	1	Thomas A et al, 2017
31	*Acmella paniculata* (Wall. ex DC.) R.K.Jansen (reported as Spilanthes calva DC.)	Asteraceae	Root	Methanol (100%) extract, formulated as a dentifrice	1	Sapra G et al., 2013
**Species Investigated only as Combination of Multiple Herbal Products**						
32	*Santalum album* L.	Santalaceae	Not stated	500 mg tablet containing 30 mg containing *Santalum album* L.	1 in combination	Shetty RN et al., 2017
33	*Prunus cerasoides* Buch.-Ham. ex D.Don	Rosaceae	Not stated	500 mg tablet containing 30 mg containing *Prunus cerasoides* Buch.-Ham. ex D.Don	1 in combination	Shetty RN et al., 2017
34	*Chrysopogon zizanioides* (L.) Roberty (reported as *Vetiveria zizanioides* (L.) Nash)	Poaceae	Not stated	500 mg tablet containing 30 mg containing *Chrysopogon zizanioides* (L.) Roberty	1 in combination	Shetty RN et al., 2017
35	*Rubia cordifolia* L.	Rubiaceae	Not stated	500 mg tablet containing 20 mg containing *Rubia cordifolia* L.	1 in combination	Shetty RN et al., 2017
36	*Woodfordia fruticosa* (L.) Kurz	Lythraceae	Not stated	500 mg tablet containing 20 mg containing *Woodfordia fruticosa* (L.) Kurz	1 in combination	Shetty RN et al., 2017
37	*Cyperus rotundus* L.	Cyperaceae	Not stated	500 mg tablet containing 20 mg containing *Cyperus rotundus* L.	1 in combination	Shetty RN et al., 2017
38	*Glycyrrhiza glabra* L.	Fabaceae	Not stated	500 mg tablet containing 10 mg containing *Glycyrrhiza glabra* L. (Munident) Oral rinse containing *G. glabra* L. extract (amount not stated)	2 in combination	Shetty RN et al., 2017 Mishra et al., 2016
39	*Berberis aristata* DC.	Berberidaceae	Not stated	500 mg tablet containing 20 mg *Berberis aristata* DC.	1 in combination	Shetty RN et al., 2017
40	*Mimosa pudica* L.	Fabaceae	Not stated	500 mg tablet containing 20 mg *Mimosa pudica* L.	1 in combination	Shetty RN et al., 2017
41	*Symplocos racemosa* Roxb.	Symplocaceae	Not stated	500 mg tablet containing 20 mg *Symplocos racemosa* Roxb.	1 in combination	Shetty RN et al., 2017
42	*Curcuma longa* L.	Zingiberaceae	Not stated	500 mg tablet containing 20 mg *Curcuma longa* L.	1 in combination	Shetty RN et al., 2017
43	*Cinnamomum verum* J. Presl (reported as *Cinnamomum zeylanicum*)	Lauraceae	Not stated	500 mg tablet containing 20 mg *Cinnamomum verum* J.Presl	1 in combination	Shetty RN et al., 2017
44	*Nardostachys jatamansi* (D.Don) DC.	Caprifoliaceae	Not stated	500 mg tablet containing 20 mg *Nardostachys jatamansi* (D.Don) DC.	1 in combination	Shetty RN et al., 2017
45	*Acorus calamus* L.	Acoraceae	Not stated	500 mg tablet containing 20 mg *Acorus calamus* L.	1 in combination	Shetty RN et al., 2017
46	*Aquilaria agallocha* Roxb.	Thymelaeaceae	Not stated	500 mg tablet containing 10 mg *Aquilaria agallocha* Roxb.	1 in combination	Shetty RN et al., 2017
47	*Syzygium aromaticum* (L.) Merr. & L.M.Perry	Myrtaceae	Not stated	500 mg tablet containing 20 mg *Syzygium aromaticum* (L.) Merr. and L.M.Perry	1 in combination	Shetty RN et al., 2017
48	*Jasminum officinale* L.	Oleaceae	Not stated	500 mg tablet containing 10 mg *Jasminum officinale L.*	1 in combination	Shetty RN et al., 2017
49	*Cinnamomum camphora* (L.) J.Presl	Lauraceae	Not stated	500 mg tablet containing 30 mg *Cinnamomum camphora* (L.) J.Presl	1 in combination	Shetty RN et al., 2017
50	*Areca catechu* L.	Arecaceae	Not stated	500 mg tablet containing *A. catechu* L. as a flavoring agent (amount not stated)	1 in combination	Shetty RN et al., 2017
51	*Piper betle* L. (reported as *Piper betel*)	Piperaceae	NA^§§^	NA (formulated as a herbal mouthwash)	1	Sharma A et al., 2018
52	*Gaultheria fragrantissima* Wall.	Ericaceae	NA^§§^	NA (formulated as a herbal mouthwash)	1	Sharma A et al., 2018
53	*Mentha × piperita* L.	Lamiaceae	NA^§§^	NA (formulated as a herbal mouthwash)	1	Sharma A et al., 2018
54	*Trachyspermum ammi* (L.) Sprague	Apiaceae	NA^§§^	NA (formulated as a herbal mouthwash)	1	Sharma A et al., 2018
55	*Aloe vera* (L.) Burm.f.	Xanthorrhoeaceae	NA^§§§^	NA (formulated as a herbal mouthwash)	1	Nandhini T et al., 2015
56	*Echinacea sp.*	Asteraceae	NA^§§§^	NA (formulated as a herbal mouthwash)	1	Nandhini T et al., 2015
57	*Hydrastis canadensis* L.	Ranunculaceae	NA^§§§^	NA (formulated as a herbal mouthwash)	1	Nandhini T et al., 2015
58	*Calendula officinalis* L.	Asteraceae	NA^§§§^	NA (formulated as a herbal mouthwash)	1	Nandhini T et al., 2015
59	*Citrus paradisi* Macfad.	Rutaceae	Seed	“Seed extract” (formulated as a herbal mouthwash)	1	Nandhini T et al., 2015
60	*Senegalia catechu* (L. f.) P.J.H. Hurter & Mabb.	Fabaceae	NA	NA (formulated as a herbal mouthwash)	1	Mishra R et al., 2016
61	*Mimusops elengi* L.	Sapotaceae	NA	NA (formulated as a herbal mouthwash)	1	Mishra R et al., 2016
62	*Ocimum tenuiflorum* L.	Lamiaceae	NA	NA (formulated as a herbal mouthwash)	1	Mishra R et al., 2016
63	*Quercus infectoria* G.Olivier	Fagaceae	NA	NA (formulated as a herbal mouthwash)	1	Mishra R et al., 2016
64	*Azadirachta indica* A.Juss.	Meliaceae	NA	NA (formulated as a herbal mouthwash)	1	Mishra R et al., 2016
65	*Syzygium aromaticum* (L.) Merr. & L.M.Perry	Myrtaceae	NA	NA (formulated as a herbal mouthwash)	1	Mishra R et al., 2016
66	*Mentha spicata* L.	Lamiaceae	NA	NA (formulated as a herbal mouthwash)	1	Mishra R et al., 2016
67	*Apium graveolens* L.	Apiaceae	NA	NA (formulated as a herbal mouthwash)	1	Mishra R et al., 2016

* Triphala is obtained by mixing equal parts of dry extracts of *Terminalia chebula*, *T. bellirica,* and *Emblica officinalis*. **, *** We could not get access to a full-text version of the work. ^§^ Hiora is a mouthwash whose ingredients are: *Salvadora persica* L., *Piper betle* L., *Terminalia bellirica* (Gaertn.) Roxb., *Gaultheria fragrantissima* Wall., *Elettaria cardamomum* (L.) Maton, *Mentha × piperita* L., *Trachyspermum ammi* (L.) Sprague. We could not identify the herbal part from each species used as an ingredient. ^§§^ The herbal ingredients of the product are only listed with the vernacular names, but the parts used are not stated. We identified the equivalent scientific names of the species in a publicly available clinical trial register (http://apps.who.int/trialsearch/Trial2.aspx?trialid=CTRI/2017/12/010895), but not the herbal parts. ^§§§^ Herbal products are not stated. The herbal mouthwash was described in tabular form as containing *Aloe vera*, "Echinaecea", "Golden seal", "Calendula", "Grapefruit seed extract" (plus excipients). *^♠^* Herboral is an alcohol-free, sugar-free product, based on triphala (see above for its composition), *Senegalia catechu* (L. f.) P.J.H. Hurter and Mabb., *Mimusops elengi* L., *Ocimum tenuiflorum* L., *Glycyrrhiza glabra* L., *Quercus infectoria* G.Olivier, *Azadirachta indica* A.Juss., *Syzygium aromaticum* (L.) Merr. and L.M.Perry, *Mentha spicata* L., *Apium graveolens* L.

## Supplementary Materials

The following are available online at https://www.mdpi.com/2218-273X/9/12/884/s1, Table S1: Non-clinical efficacy studies performed on the plant species reviewed.

## References

[1] Somaraj V., Shenoy R.P., Shenoy Panchmal G., Kumar V., Jodalli P.S., Sonde L. (2017). Effect of Herbal and Fluoride Mouth Rinses on *Streptococcus mutans* and Dental Caries among 12–15-Year-Old School Children: A Randomized Controlled Trial. Int. J. Dent..

[2] Featherstone J. (2008). Dental caries: A dynamic disease process. Aust. Dent. J..

[3] Pitts N.B., Zero D.T., Marsh P.D., Ekstrand K., Weintraub J.A., Ramos-Gomez F., Tagami J., Twetman S., Tsakos G., Ismail A. (2017). Dental caries. Nat. Rev. Dis. Primers.

[4] Mathur V.P., Dhillon J.K. (2018). Dental Caries: A Disease Which Needs Attention. Indian J. Pediatrics.

[5] Preshaw P.M., Henne K., Taylor J.J., Valentine R.A., Conrads G. (2017). Age-related changes in immune function (immune senescence) in caries and periodontal diseases: A systematic review. J. Clin. Periodontol..

[6] Villhauer A.L., Lynch D.J., Drake D.R. (2017). Improved method for rapid and accurate isolation and identification of Streptococcus mutans and Streptococcus sobrinus from human plaque samples. J. Microbiol. Methods.

[7] Banas J.A., Drake D.R. (2018). Are the mutans streptococci still considered relevant to understanding the microbial etiology of dental caries?. BMC Oral Health.

[8] Fragkou S., Balasouli C., Tsuzukibashi O., Argyropoulou A., Menexes G., Kotsanos N., Kalfas S. (2016). Streptococcus mutans, Streptococcus sobrinus and Candida albicans in oral samples from caries-free and caries-active children. Eur. Arch. Paediatr. Dent..

[9] Okada M. (2005). Longitudinal study of dental caries incidence associated with Streptococcus mutans and Streptococcus sobrinus in pre-school children. J. Med. Microbiol..

[10] Okada M., Kawamura M., Oda Y., Yasuda R., Kojima T., Kurihara H. (2012). Caries prevalence associated with Streptococcus mutans and Streptococcus sobrinus in Japanese schoolchildren: Caries prevalence in schoolchildren. Int. J. Paediatr. Dent..

[11] Oda Y., Hayashi F., Okada M. (2015). Longitudinal study of dental caries incidence associated with Streptococcus mutans and Streptococcus sobrinus in patients with intellectual disabilities. BMC Oral Health.

[12] Krzyściak W., Jurczak A., Kościelniak D., Bystrowska B., Skalniak A. (2014). The virulence of Streptococcus mutans and the ability to form biofilms. Eur. J. Clin. Microbiol. Infect. Dis..

[13] Koo H., Falsetta M.L., Klein M.I. (2013). The Exopolysaccharide Matrix: A Virulence Determinant of Cariogenic Biofilm. J. Dent. Res..

[14] Avilés-Reyes A., Miller J.H., Lemos J.A., Abranches J. (2017). Collagen-binding proteins of *Streptococcus mutans* and related streptococci. Mol. Oral Microbiol..

[15] Ito T., Ichinosawa T., Shimizu T. (2017). Streptococcal adhesin SspA/B analogue peptide inhibits adherence and impacts biofilm formation of Streptococcus mutans. PLoS ONE.

[16] Damle S.G., Loomba A., Dhindsa A., Loomba A., Beniwal V. (2016). Correlation between dental caries experience and mutans streptococci counts by microbial and molecular (polymerase chain reaction) assay using saliva as microbial risk indicator. Dent. Res. J. (Isfahan).

[17] Tanner A.C.R., Mathney J.M.J., Kent R.L., Chalmers N.I., Hughes C.V., Loo C.Y., Pradhan N., Kanasi E., Hwang J., Dahlan M.A. (2011). Cultivable Anaerobic Microbiota of Severe Early Childhood Caries. J. Clin. Microbiol..

[18] Femiano F., Femiano R., Femiano L., Jamilian A., Rullo R., Perillo L. (2016). Dentin caries progression and the role of metalloproteinases: an update. Eur. J. Paediatr. Dent..

[19] Sheiham A., James W.P.T. (2015). Diet and Dental Caries: The Pivotal Role of Free Sugars Reemphasized. J. Dent. Res..

[20] Frencken J.E., Sharma P., Stenhouse L., Green D., Laverty D., Dietrich T. (2017). Global epidemiology of dental caries and severe periodontitis—A comprehensive review. J. Clin. Periodontol..

[21] Tonetti M.S., Bottenberg P., Conrads G., Eickholz P., Heasman P., Huysmans M.-C., López R., Madianos P., Müller F., Needleman I. (2017). Dental caries and periodontal diseases in the ageing population: call to action to protect and enhance oral health and well-being as an essential component of healthy ageing—Consensus report of group 4 of the joint EFP/ORCA workshop on the boundaries be. J. Clin. Periodontol..

[22] World Health Organization What is the Burden of Oral Disease?. http://www.who.int/oral_health/disease_burden/global/en/.

[23] Pitts N.B., Zero D.T. White Paper on Dental Caries Prevention and Management. https://www.fdiworlddental.org/sites/default/files/media/documents/2016-fdi_cpp-white_paper.pdf.

[24] Flink H., Tegelberg Å., Arnetz J.E., Birkhed D. (2017). Patient-reported negative experiences related to caries and its treatment among Swedish adult patients. BMC Oral Health.

[25] WHO Collaborating Centre for Drug Statistics Methodology ATC/DDD Index. https://www.whocc.no/atc_ddd_index/?code=A01A.

[26] Kocak M.M., Ozcan S., Kocak S., Topuz O., Erten H. (2009). Comparison of the efficacy of three different mouthrinse solutions in decreasing the level of streptococcus mutans in saliva. Eur. J. Dent..

[27] Makvandi P., Jamaledin R., Jabbari M., Nikfarjam N., Borzacchiello A. (2018). Antibacterial quaternary ammonium compounds in dental materials: A systematic review. Dent. Mater..

[28] Makvandi P., Ghaemy M., Ghadiri A.A., Mohseni M. (2015). Photocurable, Antimicrobial Quaternary Ammonium-modified Nanosilica. J. Dent. Res..

[29] Jiao Y., Niu L., Ma S., Li J., Tay F.R., Chen J. (2017). Quaternary ammonium-based biomedical materials: State-of-the-art, toxicological aspects and antimicrobial resistance. Prog. Polym. Sci..

[30] Makvandi P., Ghaemy M., Mohseni M. (2016). Synthesis and characterization of photo-curable bis-quaternary ammonium dimethacrylate with antimicrobial activity for dental restoration materials. Eur. Polym. J..

[31] Makvandi P., Gu J.T., Zare E.N., Ashtari B., Moeini A., Tay F.R., Niu L.-N. (2019). Polymeric and inorganic nanoscopical antimicrobial fillers in dentistry. Acta Biomater..

[32] Bina F., Rahimi R. (2017). Sweet Marjoram: A Review of Ethnopharmacology, Phytochemistry, and Biological Activities. J. Evid. Based Complementary Altern. Med..

[33] Dinu M., Popescu M.L., Ancuceanu R., Hovaneț M.V., Ghitulescu G. (2012). Contribution to the pharmacognostical and phytobiological study on Abutilon theophrasti Medik. (Malvaceae). Farmacia.

[34] Frezza C., Venditti A., Toniolo C., Vita D., Serafini I., Ciccòla A., Franceschin M., Ventrone A., Tomassini L., Foddai S. (2019). Pedicularis L. Genus: Systematics, Botany, Phytochemistry, Chemotaxonomy, Ethnopharmacology, and Other. Plants.

[35] Badea V., Nuca C., Amariei C., Zaharia A., Bucur L.A., Grigorian M. (2013). Researches regarding the monitoring complementary treatment in periodontitis by using salivary 8-OHdG biomarker. Farmacia.

[36] Pham M.T., Rajić A., Greig J.D., Sargeant J.M., Papadopoulos A., McEwen S.A. (2014). A scoping review of scoping reviews: Advancing the approach and enhancing the consistency. Res. Synth. Methods.

[37] Arksey H., O’Malley L. (2005). Scoping studies: towards a methodological framework. Int. J. Soc. Res. Methodol..

[38] Snoussi M., Dehmani A., Noumi E., Flamini G., Papetti A. (2016). Chemical composition and antibiofilm activity of Petroselinum crispum and Ocimum basilicum essential oils against Vibrio spp. strains. Microb. Pathog..

[39] Higgins J.P.T., Sally G. (2011). Cochrane Handbook for Systematic Reviews of Interventions, Version 5.1.0. https://handbook-5-1.cochrane.org/front_page.htm.

[40] Yadav M., Kaushik M., Roshni R., Reddy P., Mehra N., Jain V., Rana R. (2017). Effect of Green Coffee Bean Extract on Streptococcus mutans Count: A Randomised Control Trial. J. Clin. Diagn. Res..

[41] European Medicines Agency List of Pharmaceutical Dosage Forms. https://www.ema.europa.eu/documents/other/list-pharmaceutical-dosage-forms_en.xls.

[42] Takenaka S., Ohsumi T., Noiri Y. (2019). Evidence-based strategy for dental biofilms: Current evidence of mouthwashes on dental biofilm and gingivitis. Jpn. Dent. Sci. Rev..

[43] Freires I.A., Rosalen P.L. (2016). How Natural Product Research has Contributed to Oral Care Product Development? A Critical View. Pharm. Res..

[44] Farah C.S., McCullough M.J., McIntosh L. (2009). Mouthwashes. Aust. Prescr..

[45] Below H., Assadian O., Baguhl R., Hildebrandt U., Jäger B., Meissner K., Leaper D.J., Kramer A. (2017). Measurements of chlorhexidine, p-chloroaniline, and p-chloronitrobenzene in saliva after mouth wash before and after operation with 0.2% chlorhexidine digluconate in maxillofacial surgery: A randomised controlled trial. Br. J. Oral Maxillofac. Surg..

[46] Cummins D., Creeth J.E. (1992). Delivery of Antiplaque Agents from Dentifrices, Gels, and Mouthwashes. J. Dent. Res..

[47] Şenel S., İkinci G., Kaş S., Yousefi-Rad A., Sargon M.F., Hıncal A.A. (2000). Chitosan films and hydrogels of chlorhexidine gluconate for oral mucosal delivery. Int. J. Pharm..

[48] Supranoto S., Slot D., Addy M., Van der Weijden G. (2015). The effect of chlorhexidine dentifrice or gel versus chlorhexidine mouthwash on plaque, gingivitis, bleeding and tooth discoloration: A systematic review. Int. J. Dent. Hyg..

[49] Twetman S., Keller M.K. (2016). Fluoride Rinses, Gels and Foams: An Update of Controlled Clinical Trials. Caries Res..

[50] Lobo P.L.D., Fonteles C.S.R., Marques L.A.R.V., Jamacaru F.V.F., da Fonseca S.G.C., de Carvalho C.B.M., de Moraes M.E.A. (2014). The efficacy of three formulations of Lippia sidoides Cham. essential oil in the reduction of salivary Streptococcus mutans in children with caries: A randomized, double-blind, controlled study. Phytomedicine.

[51] Hu C., He J., Eckert R., Wu X., Li L., Tian Y., Lux R., Shuffer J.A., Gelman F., Mentes J. (2011). Development and evaluation of a safe and effective sugar-free herbal lollipop that kills cavity-causing bacteria. Int. J. Oral Sci..

[52] Mentes J.C., Kang S., Spackman S., Bauer J. (2012). Can a Licorice Lollipop Decrease Cariogenic Bacteria in Nursing Home Residents?. Res. Gerontol. Nurs..

[53] Peters M.C., Tallman J.A., Braun T.M., Jacobson J.J. (2010). Clinical reduction of S. mutans in pre-school children using a novel liquorice root extract lollipop: a pilot study. Eur. Arch. Paediatr. Dent..

[54] Almaz M.E., Sonmez I.S., Okte Z., Oba A.A. (2017). Efficacy of a sugar-free herbal lollipop for reducing salivary Streptococcus mutans levels: A randomized controlled trial. Clin. Oral Investig..

[55] Takami K., Touyz L.Z.G., Touyz R.M. (2009). Liquorice alert. Br. Dent. J..

[56] Ly K.A., Milgrom P., Rothen M. (2008). The potential of dental-protective chewing gum in oral health interventions. J. Am. Dent. Assoc..

[57] Tangso K.J., Ho Q.P., Boyd B.J. (2015). Confectionery-based dose forms. Curr. Drug Deliv..

[58] Shamley D., Wright B. (2017). A Comprehensive and Practical Guide to Clinical Trials.

[59] Tal J. (2011). Strategy and Statistics in Clinical Trials: A Non-Statisticians Guide to Thinking, Designing, and Executing.

[60] De Aguiar A.A.A., Saliba N.A. (2004). Toothbrushing with vegetable oil: A clinical and laboratorial analysis. Braz. Oral Res..

[61] Crivelaro de Menezes T.E., Botazzo Delbem A.C., Lourencao Brighenti F., Claudia Okamoto A., Gaetti-Jardim E.J. (2010). Protective efficacy of Psidium cattleianum and Myracrodruon urundeuva aqueous extracts against caries development in rats. Pharm. Biol..

[62] Touyz L.Z., Amsel R. (2001). Anticariogenic effects of black tea (Camellia sinensis) in caries prone-rats. Quintessence Int..

[63] Ooshima T., Minami T., Aono W., Izumitani A., Sobue S., Fujiwara T., Kawabata S., Hamada S. (1993). Oolong Tea Polyphenols Inhibit Experimental Dental Caries in SPF Rats Infected with Mutatis Streptococci. Caries Res..

[64] Otake S., Makimura M., Kuroki T., Nishihara Y., Hirasawa M. (1991). Anticaries Effects of Polyphenolic Compounds from Japanese Green Tea. Caries Res..

[65] Van Meer P.J.K., Graham M.L., Schuurman H.-J. (2015). The safety, efficacy and regulatory triangle in drug development: Impact for animal models and the use of animals. Eur. J. Pharmacol..

[66] Denayer T., Stöhr T., Roy M.V. (2014). Animal models in translational medicine: Validation and prediction. Eur. J. Mol. Clin. Med..

[67] Zero D.T. (2006). Dentifrices, mouthwashes, and remineralization/caries arrestment strategies. BMC Oral Health.

[68] Featherstone J.D.B., Stookey G.K., Kaminski M.A., Faller R.V. (2011). Recommendation for a non-animal alternative to rat caries testing. Am. J. Dent..

[69] Indrayan A., Holt M.P. (2017). Concise Encyclopedia of Biostatistics for Medical Professionals.

[70] Richens A. (2001). Proof of efficacy trials: Cross-over versus parallel-group. Epilepsy Res..

[71] Sedgwick P. (2014). Before and after study designs. BMJ.

[72] Olski T.M., Lampus S.F., Gherarducci G., Saint Raymond A. (2011). Three years of paediatric regulation in the European Union. Eur. J. Clin. Pharmacol..

[73] Tse T., Williams R.J., Zarin D.A. (2009). Reporting “basic results” in ClinicalTrials.gov. Chest.

[74] Ellwood R.P., Gomez J., Goma J., Pretty I.A. (2012). Caries clinical trial methods for the assessment of oral care products in the 21st century. Adv. Dent. Res..

[75] Gupta S.K. (2011). Non-inferiority clinical trials: Practical issues and current regulatory perspective. Indian J. Pharm..

[76] European Medicines Agency ICH Topic E 10. Choice of Control Group in Clinical Trial. Note for Guidance on Choice of Control Group in Clinical Trials (CPMP/ICH/364/96). https://www.ema.europa.eu/en/documents/scientific-guideline/ich-e-10-choice-control-group-clinical-trials-step-5_en.pdf.

[77] Follmann D.A., Balakrishnan N. (2014). Primary efficacy endpoint. Methods and Applications of Statistics in Clinical Trials, Volume 1: Concepts, Principles, Trials, and Designs.

[78] European Medicines Agency Note for Guidance on Statistical Principles for Clinical Trials (CPMP/ICH/363/96). https://www.ema.europa.eu/en/documents/scientific-guideline/ich-e-9-statistical-principles-clinical-trials-step-5_en.pdf.

[79] Hall J.C. (2011). How to dissect surgical journals: VII—The concept of outcome: Journal club. ANZ J. Surg..

[80] Guyatt G.H., Cranney A., Griffith L., Walter S., Krolicki N., Favus M., Rosen C. (2002). Summary of meta-analyses of therapies for postmenopausal osteoporosis and the relationship between bone density and fractures. Endocrinol. Metab. Clin. North Am..

[81] Fleming T.R., Powers J.H. (2012). Biomarkers and surrogate endpoints in clinical trials. Statist. Med..

[82] Dilley G.J., Koerber L.G., Roche J.R. (1977). The effects of a dietary supplement of fresh oranges on the oral health of children. Asdc J. Dent. Child.

[83] Al-Marzouki S., Evans S., Marshall T., Roberts I. (2005). Are these data real? Statistical methods for the detection of data fabrication in clinical trials. BMJ.

[84] Button K.S., Ioannidis J.P.A., Mokrysz C., Nosek B.A., Flint J., Robinson E.S.J., Munafò M.R. (2013). Power failure: Why small sample size undermines the reliability of neuroscience. Nat. Rev. Neurosci..

[85] Vollmer W.M., Papas A.S., Bader J.D., Maupomé G., Gullion C.M., Hollis J.F., Snyder J.J., Fellows J.L., Laws R.L., The PACS Collaborative Research Group (2010). Design of the Prevention of Adult Caries Study (PACS): A randomized clinical trial assessing the effect of a chlorhexidine dental coating for the prevention of adult caries. BMC Oral Health.

[86] Pedrazzi V., Leite M.F., Tavares R.C., Sato S., do Nascimento G.C., Issa J.P.M. (2015). Herbal Mouthwash Containing Extracts of *Baccharis dracunculifolia* as Agent for the Control of Biofilm: Clinical Evaluation in Humans. Sci. World J..

[87] Linden A. (2013). Assessing regression to the mean effects in health care initiatives. BMC Med. Res. Methodol..

