# Supplementary material for: Clinical Trials with Herbal Products for the Prevention of Dental Caries and Their Quality: A Scoping Study
Source: Biomolecules. 2019 Dec 17;9(12):884. doi: 10.3390/biom9120884 (PMC6995540; doi:10.3390/biom9120884)
Supplement: Supplementary file 1 [file biomolecules-09-00884-s001.pdf]

Table S1. Non-clinical efficacy studies performed on the plant species reviewed

No.	Species	*In vitro* data	*In vivo* data<br>(Species and type of study)	Reference
Species investigated as single therapy				
1.	*Coffea canephora* Pierre ex A.Froehner (reported as "Coffea Robusta")	Antibacterial effect on *S. mutans* and *S. mitis*. (one study)	NA	Mehta VV, Rajesh G, Rao A, Shenoy R, B H MP. Antimicrobial Efficacy of Punica granatum mesocarp, Nelumbo nucifera Leaf, Psidium guajava Leaf and Coffea Canephora Extract on Common Oral Pathogens: An In-vitro Study. J Clin Diagn Res. 2014 Jul;8(7):ZC65-8
2.	*Baccharis dracunculifolia* DC.	Antibacterial effect on *S. mutans* (4 studies)		Leitão DP, Filho AA, Polizello AC, Bastos JK, Spadaro AC. Comparative evaluation of in-vitro effects of Brazilian green propolis and Baccharis dracunculifolia extracts on cariogenic factors of Streptococcus mutans. Biol Pharm Bull. 2004 Nov;27(11):1834-9<br><br>Freires IA, Bueno-Silva B, Galvão LC, Duarte MC, Sartoratto A, Figueira GM, de Alencar SM, Rosalen PL. The Effect of Essential Oils and Bioactive Fractions on Streptococcus mutans and Candida albicans Biofilms: A Confocal Analysis. Evid Based Complement Alternat Med. 2015;2015:871316<br><br>Freires IA, Denny C, Benso B, de Alencar SM, Rosalen PL. Antibacterial Activity of Essential Oils and Their Isolated Constituents against Cariogenic Bacteria: A Systematic Review. Molecules. 2015 Apr 22;20(4):7329-58. doi:10.3390/molecules20047329

				Pereira CA, Costa AC, Liporoni PC, Rego MA, Jorge AO. Antibacterial activity of Baccharis dracunculifolia in planktonic cultures and biofilms of Streptococcus mutans. J Infect Public Health. 2016 May-Jun;9(3):324-30
3.	*Terminalia chebula* Retz.	Effects against against *S.mutans* glucosyltransferase (1 study) and *S. mutans* (4 studies) and others (2 studies).		Mandava K, Batchu UR, Kakulavaram S, Repally S, Chennuri I, Bedarakota S, Sunkara N. Design and study of anticaries effect of different medicinal plants against S.mutans glucosyltransferase. BMC Complement Altern Med. 2019 Aug 2;19(1):197<br><br>Nayak SS, Ankola AV, Metgud SC, Bolmal UK. An in vitro study to determine the effect of Terminalia chebula extract and its formulation on Streptococcus mutans. J Contemp Dent Pract. 2014 May 1;15(3):278-82<br><br>Pratap GM, Manoj KM, Sai SA, Sujatha B, Sreedevi E. Evaluation of three medicinal plants for anti-microbial activity. Ayu. 2012 Jul;33(3):423-8<br><br>Jebashree HS, Kingsley SJ, Sathish ES, Devapriya D. Antimicrobial Activity of Few Medicinal Plants against Clinically Isolated Human Cariogenic Pathogens-An In Vitro Study. ISRN Dent. 2011;2011:541421<br><br>Jagtap AG, Karkera SG. Potential of the aqueous extract of Terminalia chebula as an anticaries agent. J Ethnopharmacol. 1999 Dec 15;68(1-3):299-306

4.	*Terminalia bellirica* (Gaertn.) Roxb.	?		
5.	*Phyllanthus emblica* L. syn. *Emblica officinalis* Gaertn.	Antibacterial effect against *S. mutans* (2 studies)		Jain I, Jain P, Bisht D, Sharma A, Srivastava B, Gupta N. Use of traditional Indian plants in the inhibition of caries-causing bacteria--Streptococcus mutans. Braz Dent J. 2015 Mar-Apr;26(2):110-5<br><br>Hasan S, Danishuddin M, Adil M, Singh K, Verma PK, Khan AU. Efficacy of E. officinalis on the cariogenic properties of Streptococcus mutans: a novel and alternative approach to suppress quorum-sensing mechanism. PLoS One. 2012;7(7):e40319
6.	*Acacia nilotica* (L.) Delile syn. *Acacia arabica* (Lam.) Willd.	Antibacterial effect against *S. mutans* and *L. casei* (ethyl acetate extract) (one study alone, and one study for a combination with *Psidium guajava*		Barad MK, Ishnava KB, Chauhan JB. Anticariogenic activity and phytochemical studies of crude extract from some Indian plant leaves. J Intercult Ethnopharmacol. 2014; 3(2):85-90
7.	*Lippia sidoides* Cham.	Antibacterial effects on *S. mutans* and *C. albicans*		Botelho MA, Nogueira NA, Bastos GM, Fonseca SG, Lemos TL, Matos FJ, Montenegro D, Heukelbach J, Rao VS, Brito GA. Antimicrobial activity of the essential oil from Lippia sidoides, carvacrol and thymol against oral pathogens. Braz J Med Biol Res. 2007 Mar;40(3):349-56<br><br>Freires IA, Bueno-Silva B, Galvão LC, Duarte MC, Sartoratto A, Figueira GM, de Alencar SM, Rosalen PL. The Effect of Essential Oils and Bioactive Fractions on Streptococcus mutans

				and Candida albicans Biofilms: A Confocal Analysis. Evid Based Complement Alternat Med. 2015;2015:871316
8.	*Stevia rebaudiana* (Bertoni) Bertoni	Caries reduction in an in vitro model on bovine teeth.<br><br>Antimicrobial effects for different extracts (1 study).		Kishta-, Derani M, Neiva GF, Boynton JR, Kim YE, Fontana M. The antimicrobial potential of stevia in an in vitro microbial caries model. Am J Dent. 2016 Apr;29(2):87-92<br><br>Gamboa F, Chaves M. Antimicrobial potential of extracts from Stevia rebaudiana leaves against bacteria of importance in dental caries. Acta Odontol Latinoam. 2012;25(2):171-5
9.	*Glycyrrhiza uralensis* Fisch.	Antibacterial effects on isolated compounds from the herbal product (one study)		Ahn SJ, Park SN, Lee YJ, Cho EJ, Lim YK, Li XM, Choi MH, Seo YW, Kook JK. In vitro antimicrobial activities of 1-methoxyficifolinol, licorisoflavan A, and 6,8-diprenylgenistein against Streptococcus mutans. Caries Res. 2015;49(1):78-89
10.	*Bertholletia excelsa* Bonpl.	?		NA
11.	*Magnolia officinalis* Rehder & E.H.Wilson	Inhibitory effect of magnolol on glucosyltransferase (one study)<br><br>Inhibitory effect on salivary bacteria for magnolia bark extract (one study) and on S. mutans for magnolol and honokiol (one study)		Huang BB, Fan MW, Wang SL, Han DX, Chen Z, Bian Z. The inhibitory effect of magnolol from Magnolia officinalis on glucosyltransferase. Arch Oral Biol. 2006; 51(10):899-905<br><br>Greenberg M, Urnezis P, Tian M. Compressed mints and chewing gum containing magnolia bark extract are effective against bacteria responsible for oral malodor. J Agric Food Chem. 2007 Nov 14;55(23):9465-9<br><br>Sakaue Y, Domon H, Oda M, Takenaka S, Kubo M, Fukuyama Y, Okiji T, Terao Y. Anti-biofilm and bactericidal effects of magnolia bark-derived magnolol and honokiol on Streptococcus mutans. Microbiol Immunol. 2016 Jan;60(1):10-6

12.	*Camellia sinensis* (L.) Kuntze	Antimicrobial effects (for "tea" or its components) on *S. mutans* and biofilm inhibition (7 studies), L. acidophilus (1 study) and other cariogenic bacteria (2 studies)	Assessment of anticariogenic effects of tea in Sprague-Dawley rats (1 study)<br><br>Anticariogenic effect shown for oolong tea in SPF rats (1 study)<br><br>Anticariogenic effects of tea polyphenols demonstrated in rats infected with S. mutans and fed a cariogenic diet (1 study).	George DE, Shetty R, Shetty PJ, Gomes LA. An In vitro Study to Compare the Effect of Different Types of Tea with Chlorhexidine on Streptococcusmutans. J Clin Diagn Res. 2017 Sep;11(9):ZC05-ZC07<br><br>Kawarai T, Narisawa N, Yoneda S, Tsutsumi Y, Ishikawa J, Hoshino Y, Senpuku H. Inhibition of Streptococcus mutans biofilm formation using extracts from Assam tea compared to green tea. Arch Oral Biol. 2016; 68:73-82<br><br>Anita P, Sivasamy S, Madan Kumar PD, Balan IN, Ethiraj S. In vitro antibacterial activity of Camellia sinensis extract against cariogenic microorganisms. J Basic Clin Pharm. 2014 Dec;6(1):35-9<br><br>Araghizadeh A, Kohanteb J, Fani MM. Inhibitory activity of green tea (Camellia sinensis) extract on some clinically isolated cariogenic and periodontopathic bacteria. Med Princ Pract. 2013;22(4):368-72<br><br>Subramaniam P, Eswara U, Maheshwar Reddy KR. Effect of different types of tea on Streptococcus mutans: an in vitro study. Indian J Dent Res. 2012; 23(1):43-8<br><br>Naderi NJ, Niakan M, Kharazi Fard MJ, Zardi S. Antibacterial activity of Iranian green and black tea on streptococcus mutans: an in vitro study. J Dent (Tehran). 2011 Spring;8(2):55-9

				Hassani AS, Amirmozafari N, Ordouzadeh N, Hamdi K, Nazari R, Ghaemi A. Volatile components of Camellia sinensis inhibit growth and biofilm formation of oral streptococci in vitro. Pak J Biol Sci. 2008;11(10):1336-41<br><br>Touyz LZ, Amsel R. Anticariogenic effects of black tea (Camellia sinensis) in caries prone-rats. Quintessence Int. 2001 Sep;32(8):647-50<br><br>Ooshima T, Minami T, Aono W, Izumitani A, Sobue S, Fujiwara T, Kawabata S, Hamada S. Oolong tea polyphenols inhibit experimental dental caries in SPF rats infected with mutans streptococci. Caries Res. 1993;27(2):124-9<br><br>Otake S, Makimura M, Kuroki T, Nishihara Y, Hirasawa M. Anticaries effects of polyphenolic compounds from Japanese green tea. Caries Res. 1991;25(6):438-43
13.	*Salvadora persica* L.	Effect of extracts against *S. mutans* (6 studies) and other common oral pathogens (6 studies)<br><br>Inhibition of dextransucrase activity in *S. mutans* (1 study)<br><br>Cytotoxic potential assessed on human		Balto H, Al-Sanie I, Al-Beshri S, Aldrees A. Effectiveness of Salvadora persica extracts against common oral pathogens. Saudi Dent J. 2017 Jan;29(1):1-6<br><br>Siddeeqh S, Parida A, Jose M, Pai V. Estimation of antimicrobial Properties of Aqueous and Alcoholic Extracts of Salvadora Persica (Miswak) on Oral Microbial Pathogens - An Invitro Study. J Clin Diagn Res. 2016 Sep;10(9):FC13-FC16

		gingival fibroblast cells (1 study)		El-Latif Hesham A, Alrumman SA. Antibacterial activity of Miswak Salvadora persica extracts against isolated and genetically identified oral cavity pathogens. Technol Health Care. 2016 Apr 29;24 Suppl 2:S841-8<br><br>Naseem S, Hashmi K, Fasih F, Sharafat S, Khanani R. In vitro evaluation of antimicrobial effect of miswak against common oral pathogens. Pak J Med Sci. 2014 Mar;30(2):398-403<br><br>Al-Sohaibani S, Murugan K. Anti-biofilm activity of Salvadora persica on cariogenic isolates of Streptococcus mutans: in vitro and molecular docking studies. Biofouling. 2012;28(1):29-38<br><br>Sofrata AH, Claesson RL, Lingström PK, Gustafsson AK. Strong antibacterial effect of miswak against oral microorganisms associated with periodontitis and caries. J Periodontol. 2008 Aug;79(8):1474-9<br><br>Goyal D, Sharma S, Mahmood A. Inhibition of dextransucrase activity in Streptococcus mutans by plant phenolics. Indian J Biochem Biophys. 2013 Feb;50(1):48-53<br><br>Balto HA, Al-Manei KK, Bin-Mohareb TM, Shakoor ZA, Al-Hadlaq SM. Cytotoxic effect of Salvadora persica extracts on human gingival fibroblast cells. Saudi Med J. 2014; 35(8):810-5

14.	*Prunus dulcis* (Mill.) D.A.Webb	?	?	NA
15.	*Garcinia mannii* Oliv.	?	?	NA
16	*Citrus sinensis* (L.) Osbeck	Effect of orange juice on enamel demineralization assessed *in situ*.		Issa AI, Toumba KJ, Preston AJ, Duggal MS. Comparison of the effects of whole and juiced fruits and vegetables on enamel demineralisation in situ. Caries Res. 2011;45(5):448-52
17	*Scutellaria baicalensis* Georgi	?		?
18	*Psidium cattleianum* Afzel. ex Sabine	Effects of extracts on *S. mutans* viability, biofilm, acid production and protein expression (1 study)	Anticariogenic effect assessed in Wistar rats infected with S. mutans and fed with a cariogenic diet (1 study)	Brighenti FL, Luppens SB, Delbem AC, Deng DM, Hoogenkamp MA, Gaetti-Jardim E Jr, Dekker HL, Crielaard W, ten Cate JM. Effect of Psidium cattleianum leaf extract on Streptococcus mutans viability, protein expression and acid production. Caries Res. 2008;42(2):148-54<br><br>Crivelaro de Menezes TE, Botazzo Delbem AC, Lourenção Brighenti F, Cláudia Okamoto A, Gaetti-Jardim E Jr. Protective efficacy of Psidium cattleianum and Myracrodruon urundeuva aqueous extracts against caries development in rats. Pharm Biol. 2010 Mar;48(3):300-5
19	*Vaccinium macrocarpon* Aiton	Antibacterial effects on *S. mutans* and other cariogenic bacteria for the juice or different chemical components/fractions (2 studies in PubMed, 5 studies cited by L. Bonifait and D. Grenier, 2010)		Gregoire S, Singh AP, Vorsa N, Koo H. Influence of cranberry phenolics on glucan synthesis by glucosyltransferases and Streptococcus mutans acidogenicity. J Appl Microbiol. 2007; 103(5):1960-8<br><br>Koo H, Nino de Guzman P, Schobel BD, Vacca Smith AV, Bowen WH. Influence of cranberry juice on glucan-mediated processes involved in Streptococcus mutans biofilm development. Caries Res. 2006;40(1):20-7

		Inhibition of surface-adsorbed glucosyltransferases B and C and F-ATPases and acid production disruption (1 study)<br><br>Anti-adhesion effects on *C. albicans* (1 study)		Bonifait L, Grenier D. Cranberry polyphenols: potential benefits for dental caries and periodontal disease. J Can Dent Assoc. 2010;76:a130<br><br>Girardot M, Guerineau A, Boudesocque L, Costa D, Bazinet L, Enguehard-Gueiffier C, Imbert C. Promising results of cranberry in the prevention of oral Candida biofilms. Pathog Dis. 2014 Apr;70(3):432-9
20	*Allium sativum* L.	Inhibitory effects on *S. mutans* (5 studies), L. acidophilus (2 studies), *C. albicans* (1 study)		Kshirsagar MM, Dodamani AS, Karibasappa GN, Vishwakarma PK, Vathar JB, Sonawane KR, Jadhav HC, Khobragade VR. Antibacterial activity of garlic extract on cariogenic bacteria: An in vitro study. Ayu. 2018 Jul-Sep;39(3):165-168<br><br>Mathai K, Anand S, Aravind A, Dinatius P, Krishnan AV, Mathai M. Antimicrobial<br>Effect of Ginger, Garlic, Honey, and Lemon Extracts on Streptococcus mutans. J<br>Contemp Dent Pract. 2017 Nov 1;18(11):1004-1008<br><br>Thomas A, Thakur S, Mhambrey S. Comparison of the antimicrobial efficacy of chlorhexidine, sodium fluoride, fluoride with essential oils, alum, green tea, and garlic with lime mouth rinses on cariogenic microbes. J Int Soc Prev Community Dent. 2015 Jul-Aug;5(4):302-8

				Jain I, Jain P, Bisht D, Sharma A, Srivastava B, Gupta N. Use of traditional Indian plants in the inhibition of caries-causing bacteria--Streptococcus mutans. Braz Dent J. 2015 Mar-Apr;26(2):110-5<br><br>Fani MM, Kohanteb J, Dayaghi M. Inhibitory activity of garlic (Allium sativum) extract on multidrug-resistant Streptococcus mutans. J Indian Soc Pedod Prev Dent. 2007; 25(4):164-8
21	*Foeniculum vulgare* Mill.	Inhibitory effects of essential oil on *S. mutans* and *L. casei* (1 study)		Wiwattanarattanabut K, Choonharuangdej S, Srithavaj T. In Vitro Anti-Cariogenic Plaque Effects of Essential Oils Extracted from Culinary Herbs. J Clin Diagn Res. 2017 Sep;11(9):DC30-DC35
22	*Sesamum indicum* L.	?		NA
23	*Cocos nucifera* L.	?		NA
24	*Ocimum tenuiflorum* L. (reported as "*Ocimum sanctum* L.")	Inhibitory effects against *S. mutans* (2 studies) and *S. sanguis* (1 study)		Kochikar Pai R, Bhat SS, Salman A, Chandra J. Use of an Extract of Indian Sacred Plant Ocimum sanctum as an Anticariogenic Agent: An in vitro Study. Int J Clin Pediatr Dent. 2015; 8(2):99-101<br><br>Joycharat N, Limsuwan S, Subhadhirasakul S, Voravuthikunchai SP, Pratumwan S, Madahin I, Nuankaew W, Promsawat A. Anti-Streptococcus mutans efficacy of Thai herbal formula used as a remedy for dental caries. Pharm Biol. 2012; 50(8):941-7
25	*Plantago lanceolata* L.	Antimicrobial activity against cariogenic bacteria (*Streptococci, Lactobacili*) (1 study)		Ferrazzano GF, Cantile T, Roberto L, Ingenito A, Catania MR, Roscetto E, Palumbo G, Zarrelli A, Pollio A. Determination of the in vitro and in vivo antimicrobial activity on salivary Streptococci and Lactobacilli and chemical characterisation of

				the phenolic content of a Plantago lanceolata infusion. Biomed Res Int. 2015;2015:286817
26	*Pistacia lentiscus* L.	Effect against different oral pathogens of mastic or *P. lentiscus* components (e.g. oleanolic acid) (2 studies)		Karygianni L, Cecere M, Argyropoulou A, Hellwig E, Skaltsounis AL, Wittmer A, Tchorz JP, Al-Ahmad A. Compounds from Olea europaea and Pistacia lentiscus inhibit oral microbial growth. BMC Complement Altern Med. 2019 Feb 26;19(1):51<br><br>Aksoy A, Duran N, Koksal F. In vitro and in vivo antimicrobial effects of mastic chewing gum against Streptococcus mutans and mutans streptococci. Arch Oral Biol. 2006 Jun;51(6):476-81
27	*Elettaria cardamomum* (L.) Maton	?	?	
28	*Eugenia uniflora* L.	Inhibitory effect on *Streptococcus oralis* (ATCC 10557) and *Lactobacillus casei* for a dentifrice containing an alcoholic exract		Jovito VC, Freires IA, Almeida LF, Moura D, Castro RD, Paulo MQ, Leite-Cavalcanti A. Activity of in vitro forms of dentifrices containing the hydroalcoholic extract of the ripe fruit of Eugenia uniflora L. (Surinam cherry) on cariogenic bacteria. Acta Odontol Latinoam. 2011;24(1):41-6
29	*Dysphania ambrosioides* (L.) Mosyakin & Clemants (reported as *Chenopodium ambrosioides* L.)	?		NA

30	*Citrus sp.* (reported only as "lime")	?		NA
31	*Acmella paniculata* (Wall. ex DC.) R.K.Jansen (reported as *Spilanthes calva* DC.)	?		NA
